# Microbiome-immune crosstalk in allergic rhinitis: lung and intestinal microbiota mechanisms

**DOI:** 10.3389/fmicb.2025.1697226

**Published:** 2025-12-03

**Authors:** Chendong Wang, Chuntao Zhai, Shouxi Hu, Yu’e Lü

**Affiliations:** 1 The Second Clinical Medical College of Shanxi University of Chinese Medicine, Taiyuan, Shanxi Province, China; 2 Shanxi Society of Geriatrics, Shanxi Provincial Acupuncture Hospital, Taiyuan, Shanxi Province, China

**Keywords:** allergic rhinitis, lung microbiota, intestinal microbiota, immune response, microbiome-immune crosstalk

## Abstract

Allergic rhinitis (AR) is a prevalent immune-mediated upper respiratory disorder that manifests as an itchy nose, nasal congestion, a runny nose, and other symptoms. Emerging research suggests that AR, beyond its IgE-mediated hypersensitivity, indirectly influences the immune system by altering the equilibrium of the lung and intestinal microbiota. Therefore, future research should systematically characterize the mechanistic involvement of the respiratory and intestinal microbiomes in AR development, which may reveal innovative therapeutic targets. This review highlights the mechanisms by which the lung and intestinal microbiota contribute to the pathogenesis of AR and discusses potential therapeutic strategies.

## 1 Introduction

Since the 21st century, due to the combined influence of environmental and genetic factors, about 40% of the world’s population is affected by AR (Yuan et al., 2022), and about 250 million people in China suffer from it. And this condition also potentially shows progressively rising prevalence rates across populations. AR imposes a substantial medical and socio-economic burden. For instance, a 2020 survey in Beijing, China, reported an annual per capita cost of 1539.0 RMB for AR patients (Xian Li et al., 2022). AR can lead to inefficiency at work and sleep problems. Therefore, it is at the same time a global health problem that places a huge burden on the world.

AR persists as a ubiquitous allergic disorder in clinical practice, driven by the immune system’s overreaction to exogenous allergens. It is a chronic inflammatory reactive malady of the nasal mucosa. It is principally driven by the secretion of IgE-dependent substance (primarily histamine) after exposure of susceptible individuals to an allergen, with the participation of multiple immunocompetent cell populations and inflammatory mediators (Gao et al., 2024). Its typical symptoms include an itchy nose, severe rhinorrhea, and nasal obstruction, which may also be in conjunction with eye clinical manifestations, notably ocular pruritus and conjunctival congestion.

The lung and intestinal microbiota refer to the collective communities of trillions of microorganisms residing in the human respiratory and gastrointestinal tracts, respectively. They form a symbiotic relationship with the human body, involved in digestion and absorption, immune regulation, and other key physiological processes, known as the human body’s “second brain.” As research deepens, there is growing evidence that AR is not limited to the immune response in the upper respiratory tract, but that it is tightly bound to variations in lung and intestinal microbiota (Zhou et al., 2021; Weronika et al., 2020). The modulatory effect of the microbiota on the host defense system—immune system, especially the interactions in the lungs and intestine, may provide new perspectives on the pathogenesis of AR. We also make some related hypotheses about the possibility that the intestinal-lung axis, which may serve as a bridge between the lungs and the intestine, may be a relevant possibility. In this paper, we review the relevant literature and provide an overview of the mechanistic connection between this disease and the intestinal-lung microbiota grounded in the latest national and international advances. We provide some innovative ideas for clinical treatment to deepen the understanding of AR and to lay the foundation for future clinical diagnosis, treatment, and prognosis for a large sample of patients.

## 2 Mechanistic contribution of lung microbiota in AR

Although the presence of microorganisms has been known to humans for more than three centuries (Clifford and Antony, 1932), in the absence of clinical manifestations, the lungs have long been thought to be sterile. Nowadays, it is gradually accepted that healthy lungs also contain microbiota (Dickson et al., 2015; Natalini et al., 2023), but the impact of lung microbiota on the local immune system of the lungs is still open to debate (Segal and Blaser, 2014; Dickson et al., 2015).

In any case, the lung microbiota plays an integral role in the pathogenesis of AR. The lungs, as vital organs, are also rich in microbiota. The nasopharynx is connected to the lungs, which are part of the respiratory system, and pathologically, diseases of the lungs often affect the nasopharynx; therefore, nasopharyngeal microorganisms are included in this review.

### 2.1 Differences in lung microbiota between normal subjects and AR patients

It has been shown that the microorganisms of the inferior turbinate in patients with Sudden worsening AR consisted primarily of the *Proteobacteria* phylum, phylum *Firmicutes,* and *Bacteroidete* phylum, similar to healthy controls (HC; Yuan et al., 2022), but differed in that the *Actinobacteria* phylum was significantly increased in AR (Charlotte et al., 2021; *p* < 0.05). The specific bacterial species involved require further identification and characterization. In addition, under typical circumstances, the microbiota of the nasopharynx is dominated by the *Proteus* (Such as *Moraxella* and *Haemophilus genus*), phylum *Firmicutes* (Such as *Staphylococcus* and *Candida albicans*), and the *Actinobacteria* phylum (Charlotte et al., 2021; Massimiliano et al., 2020). The variation of these genera in AR versus healthy populations suggests their potential to serve as diagnostic markers or to provide a macroscopic basis for further mechanistic studies.

### 2.2 Mechanisms of lung microbiome dysbiosis in AR

Specific groups of lung bacteria, such as the *Firmicutes*, *Bacteroidetes*, and *Proteobacteria*, are associated with susceptibility and severity of AR (Yuan et al., 2022). These phenomena signal a dysbiosis of the lung microbiota in patients who have AR symptoms. While dysbiosis of the lung microbiota may trigger or exacerbate the symptoms of AR by modulating the immune response. The mechanism may be that the metabolites of these bacteria enable Th cells to differentiate into cells with different functions according to the nature of the antigen, microenvironmental cytokines, and the regulation of antigen-presenting cells (Zhu et al., 2010), exemplified by Th17 cells. And it stimulates numerous T lymphocytes and macrophages present in the interstitium of the lungs, thus promoting their uptake of antigens as well as the release of IgE, which ultimately exacerbates AR. It is noteworthy that Th cells can also differentiate into Th9 (which can release anti-inflammatory mediators such as IL-10 and thus inhibit the inflammatory response). This suggests that it becomes a question of how to modulate the lung microbiota and the environmental conditions in which they are located in order to target and regulate the differentiation of Th cells and thus promote or inhibit AR.

#### 2.2.1 Mechanism of *Staphylococcus aureus* in the lung affecting AR

Ecological imbalance in pulmonary microbiota, in addition to affecting local immune regulation, can also affect the IgE immune response, thus leading to exacerbation of AR. Certain pulmonary bacterial metabolites or lysates can affect IgE production, thereby promoting AR. For example, a study described lower microbial species but higher *Staphylococcus aureus* abundance up to 80%, whereas low IgE levels of *S. aureus* were almost zero in patients who had higher serum IgE (≥17.5 IU/ml) versus patients who had lower IgE (Hyun et al., 2018; 0 to 0.69 IU/ml). *Staphylococcus aureus* triggers IgE synthesis and exacerbates AR through a mechanism whereby superantigens (SAgs) released by the *Staphylococcus aureus* directly activate T cells by linking T cell receptors to class II major histocompatibility complex molecules on antigen-presenting cells. This leads to massive release of Th2 cytokines (IL-4, IL-5, and IL-13), thereby enhancing eosinophilic inflammation and IgE production (Sim et al., 2024). Interestingly, however, IgE exceeding clinical thresholds can in turn lead to *Staphylococcus aureus* proliferation, activating mast cell granule exocytosis and causing inflammation (Yuan et al., 2022). Unfortunately, research into the underlying mechanisms remains extremely limited. Current human studies have not definitively confirmed that IgE directly stimulates *Staphylococcus aureus* proliferation. However, one possible explanation is that the mechanism may involve *Staphylococcus aureus* lysate, under conditions of high IgE levels, potentially activating immune cells such as mast cells to release certain cytokines. This alters the local microenvironment, indirectly creating conditions favorable for *Staphylococcus aureus* proliferation—conditions that may require specific cytokines secreted by immune cells like mast cells. Another possibility is that *Staphylococcus aureus* binds to IgE via surface proteins, leveraging IgE’s immunomodulatory properties to enhance its own adhesion capabilities, thereby facilitating easier colonization within the host. And the increased colonization facilitates *Staphylococcus aureus*’s acquisition of nutrients from human cells, thereby sustaining its proliferation.

Consequently, targeted reduction of *Staphylococcus aureus* colonization and IgE titers represents a promising therapeutic approach for AR (Charlotte et al., 2021). The above findings also indicate that *Staphylococcus aureus* and IgE can mutually enhance each other. Therefore, for patients who are unsuitable for antibiotic therapy and where *Staphylococcus aureus* is difficult to control, selecting IgE antagonists to reduce the bacterium’s adhesion capacity represents an effective strategy for controlling *Staphylococcus aureus*.

#### 2.2.2 Mechanism of *Streptococcus salivarius* in the lung affecting AR

*Streptococcus salivarius* is a major bacterial component of the human oral cavity and upper respiratory tract (Nakajima et al., 2014; Miao et al., 2023). It exacerbates the pathogenesis of AR by adhering to nasal epithelial cells, triggering pro-inflammatory cytokine secretion, and inducing structural remodeling of the nasal mucosa (Miao et al., 2023). The mechanism involves the adhesion of *Streptococcus salivarius* pro-inflammatory factors to Muc5ac mucin on the surface of nasal epithelial cells. Following adhesion, the interaction between bacterial surface virulence factors and host epithelial receptors amplifies the inflammatory response (Miao et al., 2023). A randomized controlled trial involving human subjects has demonstrated that under specific conditions, *Streptococcus salivarius* isolates consistently stimulate pro-inflammatory cytokines IL-6, IL-8, TNF-α, epithelial cytokines IL-33 and TSLP (triggering Th2-mediated allergic cascades that exacerbate AR), and the expression of the chemokine CCL11 (which specifically recruits eosinophils via chemotaxis, leading to their accumulation and adhesion in the nasal cavity, where they release inflammatory mediators like histamine, thereby worsening AR; Miao et al., 2023). Experimental evidence from both *in vitro* systems and AR animal models demonstrates that *Streptococcus salivarius* directly exacerbates hallmark AR symptoms, establishing an etiological role in disease pathogenesis.

Theoretically, interventions targeting the interaction between *Streptococcus salivarius* and Muc5ac mucin on the surface of nasal epithelial cells, such as blocking bacterial adhesion factors, could represent a novel therapeutic strategy.

### 2.3 A short summary of the therapeutic potential

The lung microbiota critically regulates the pathogenesis of AR. Clinical control of AR can be achieved by testing the abundance of *Firmicutes* and *Proteobacteria*, or by taking medications containing these organisms for prevention. The use of antibiotics to kill *Staphylococcus aureus* effectively reduces the number of *Staphylococcus aureus*, thus relieving AR, and for patients for whom antibiotics are not appropriate, IgE antagonists can also be used for treatment. Antibiotics can also be used to control the *Bacteroides*, thus relieving the symptoms of AR. *Streptococcus salivarius* exacerbates the development of AR, suggesting that in the future, targeted therapies could also be achieved by controlling nasal *Streptococcus salivarius* populations, as well as by targeting and blocking the binding of the Muc5ac mucin to *Streptococcus salivarius* pro-inflammatory factors, thereby preventing an increase in the inflammatory response.

## 3 The mechanistic contribution of intestinal microbiota in AR

The intestinal microbiota is one of the richest ecosystems of microbiota in human physiology, and it is indispensable for the regulation of the immune system. Patients with AR exhibit consistent signatures of an ecological dysregulation in the intestinal microbiota (Zhou et al., 2021), such as a decrease in beneficial microbiota along with an increase in pathogenic microbiota. Below are specific alterations in intestinal bacteria in patients who have AR symptoms and their possible mechanisms. Intestinal bacteria have many effects on the development of AR, and some of them seem to have antagonistic effects on each other. The relationship between AR and microorganisms has been studied, and by elaborating on these relationships, some guidelines for the treatment of AR have been suggested.

### 3.1 Disparities in intestinal microorganisms between healthy controls and AR-affected individuals

There have been several studies showing that some bacteria are significantly different between AR patients and normal people. Notably, AR patients showed reduced microbiota diversity, featuring increased *Bacteroides* but reduced *Actinobacteria* and *Proteobacteria* (XiangTao et al., 2020; *p* < 0.001, q < 0.001). However, as a further illustration, OVA-induced AR mice exhibited elevated *Proteobacteria* alongside reduced *Bacteroides* and *Actinobacteria* proportions (Kim et al., 2019). These observed discrepancies may be attributed to inherent physiological differences between humans and mice, as well as variations in microbial taxa. Understanding the disparities in the microbiota between AR patients and healthy controls and the effects of these disparities helps us in diagnostic treatment. Substantial additional research is required to elucidate the underlying mechanisms of these observations and thus to provide evidence for clinical treatment.

#### 3.1.1 Role of bifidobacteria in AR

Members of the *Bifidobacteria* genus (*Actinobacteria*) are anaerobic, non-flagellated, Gram-positive bacilli that typically inhabit mammalian digestive systems, reproductive tracts, and oral ecosystems. Some researchers have investigated the role of oral *Bifidobacteria* breve in allergic rhinitis, observing that it exerts anti-allergic effects by suppressing Th2-type immune responses and enhancing CD4 + CD25 + Treg cell activity. A random-effects model indicates that early supplementation with probiotics, including *Bifidobacteria* breve, can reduce the risk of atopic sensitization in children and decrease total IgE levels, thereby alleviating AR (Von and Laubach, 2014; Yin et al., 2019).

However, after analysing the studies, we can see that *Bifidobacteria* have a major inhibitory effect on AR, and the main way to achieve this effect is to inhibit inflammation by suppressing the immune response of Th2 cells. And the *Bifidobacteria* found in the study had a promoting effect on Treg cells. The specific mechanism involves the cell wall peptidoglycan of *Bifidobacteria* being recognized by Toll-like receptors (TLRs) on the surface of immune cells such as dendritic cells or macrophages, prompting these immune cells to secrete IL-12 (Li et al., 2000). Subsequently, IL-12 promotes the differentiation of naive T cells into Th1 cells and stimulates these Th1 cells to secrete gamma interferon (IFN-γ; Krueger et al., 2021). IFN-γ ultimately directly inhibits the proliferation and function of Th2 cells, suppressing their ability to secrete pro-inflammatory cytokines, thereby alleviating AR.

*Bifidobacteria* can also activate plasma cells under the intestinal epithelium to synthesize secretory IgA (Maozhen et al., 2024), which can play a protective role against endogenous commensal bacteria as well as external pathogens and other harmful substances on the body, and help to protect the organism in good health. The mechanism can be summarized as follows: antigenic substances on the surface of *Bifidobacteria* are recognized by Toll-like receptors (TLRs) on the surface of dendritic cells within the intestine. Subsequently, the dendritic cells are activated and produce signaling molecules (Verma et al., 2018) such as TGF-β. TGF-β is a cytokine capable of inducing secretory IgA (sIgA) secretion from surface IgA cells (a subtype of B cells; Lebman et al., 1990). sIgA is released into the intestinal lumen, where it can coat bacteria and harmful substances, preventing their adhesion to the intestinal wall and thereby suppressing intestinal inflammation.

Given that sIgA can encapsulate bacteria, thereby neutralizing the pro-inflammatory effects of certain pro-inflammatory bacteria—meaning these bacteria lose their pro-inflammatory capacity due to encapsulation—this aligns with the outcome of suppressed proliferation of pro-inflammatory intestinal microbes, though the mechanisms differ. Furthermore, the absence of certain pro-inflammatory intestinal bacteria also constitutes a key mechanism promoting AR remission. Therefore, we have reason to speculate that sIgA may remotely alleviate AR by coating bacteria that remotely promote AR, thereby rendering them ineffective. Clinical experiments could be conducted to determine medical reference values for *Bifidobacteria* abundance, which would be promising for intervening in allergic diseases by regulating the abundance of *Bifidobacteria*.

#### 3.1.2 The role of the balance between *Staphylococcus aureus* and the commensal bacterium *Clostridium* in AR

*Staphylococcus aureus* colonized in the ileal epithelium induces IL-22 production by ILC3 (type 3 intrinsic lymphocytes), which consequently upregulates epithelial-derived serum amyloid A1 and A2. Thereby promoting the development of Th17 (whose main role can be summarized as secreting cytokines to stimulate the upregulation of pro-inflammatory cytokine expression by various types of cells, recruiting neutrophils to form an inflammatory response) cells. In addition, *Staphylococcus aureus* enterotoxins act as superantigens (superantigens are a class of specialized antigens capable of bypassing the conventional antigen presentation process and directly activating a large number of T cells) that induce Th2 cell differentiation (Jin et al., 2023), IgE class-switching, and eosinophil-dominated inflammation (Sim et al., 2024). In contrast, commensal *Clostridium* species indirectly promote the development of regulatory T cells (Tregs) and IL-10 production by CD4 + T cells through the generation of SCFAs, thereby suppressing immune responses (Yang et al., 2020). When the ratio of the above microbiota is abnormal, it can indirectly lead to an imbalance between Th17 and Treg and allergies. Therefore, clinical treatment of AR can be carried out by regulating the ratio of *Staphylococcus aureus* to the commensal bacterium *Clostridium*. However, the medical reference values for the counts of the two genera must be determined previously, and a large number of relevant experiments still need to be done in the clinic.

In addition, some research described that individuals with higher total serum IgE had lower microbial species but higher abundance of *Staphylococcus aureus* compared to those with lower IgE (Hyun et al., 2018). This demonstrated a significant positive association was observed between *Staphylococcus aureus* colonization and serum IgE concentrations. Therefore, reducing *Staphylococcus aureus* abundance within defined limits and IgE levels in the intestine might serve as a promising treatment modality for the prevention of IgE-associated diseases, including AR.

#### 3.1.3 *Prevotella* and AR

*Prevotella* is essentially the most plentiful intestinal microbiota (Yuan et al., 2022), and *Prevotella* are significantly increased in patients with AR One experiment used random forests and performed 10-fold cross-validation on the model. A receiver operating characteristic (ROC) curve was plotted to evaluate the predictive ability, with an area under the curve (AUC) of 0.9628 (95% CI: 0.906–1.000). Specific data figures are referenced in the literature (Costello et al., 2009). This is because *Prevotella* bacteria can produce short-chain fatty acids (SCFAs) with anti-inflammatory properties from dietary fiber, thereby providing corresponding defense mechanisms in AR patients, leading to increased levels in this population. The effects of downstream SCFAs on AR will be detailed in Chapter 8, “Metabolites.”

The link between *Prevotella* and diet—particularly the high-fat, low-fiber Western diet—could account for its decline in Western populations. This suggests that eating a diet rich in fat and fiber may reduce the colonization of *Prevotella*, thereby decreasing the body’s immunity to AR. The primary reason is likely that *Prevotella* bacteria excel at breaking down complex polysaccharides (fiber) in plants as their main energy source. Consequently, a low-fiber diet suppresses *Prevotella* proliferation. Patients on high-fat, high-protein diets often experience reduced energy demands from carbohydrates (especially dietary fiber), leading to decreased intake. This, in turn, reduces the energy substrate available to *Prevotella*, inhibiting its proliferation and exacerbating AR.

These are the common microorganisms and their interrelationships that have an impact on allergic diseases, which can be controlled by probiotics or medications that work to increase the beneficial bacteria and decrease the harmful ones. For example, (1) By transplantation of *Bifidobacteria*, the immune response of Th2 cells can be inhibited to suppress inflammation. (2) Regulation of the ratio of *Staphylococcus aureus* (pro-inflammatory bacteria) to the commensal bacteria *Clostridium* (anti-inflammatory bacteria), also by means of microbial transplantation. (3) Through selective transplantation of *Prevotella* bacteria. These may become new therapeutic targets in the future.

Comparison of lung and intestinal microbiota, metabolites, and related mechanisms in AR patients is shown in Table 1.

**Table 1:** Comparative analysis of microbiota, metabolites, and mechanisms in AR patients.

(A) Microbial alterations			
Location	Increased in number in AR	Decreased in number in AR	Reference
Lung	*Staphylococcus aureus*	-	Hyun et al. (2018)
	*Streptococcus salivarius*	-	Miao et al. (2023)
Intestinal	*Staphylococcus aureus*	Symbiotic bacterium *Clostridium*	Jin et al. (2023); Sim et al. (2024); Yang et al., 2020
	-	*Bacteroides*	Kim et al. (2019)
	-	*Bifidobacterium*	Von and Laubach (2014); Yin et al. (2019)
	*Prevotella*	*-*	Costello et al. (2009)

(B) Key metabolites			
Location	Metabolites	Change	Reference
Lung	*Staphylococcus aureus* lysates and IgE	Increase	Hyun et al. (2018); Sim et al. (2024)
	IL-6, IL-8, TNF-α and CCL11	Increase	Miao et al. (2023)
Intestinal	IL-22 and serum amyloid A1 and A2	Increase	-
	IL-10 and IgA	Dcrease	Maozhen et al. (2024); Yang et al. (2020)

(C) Underlying mechanisms		
Location	Mechanism Summary	Reference
Lung	*Staphylococcus aureus* → IgE secretion	Sim et al. (2024)
	*Streptococcus salivarius* → IL-6, IL-8, TNF-α CCL11 → eosinophils chemotaxis→inflammatory	Miao et al. (2023)
Intestial	*Staphylococcus aureus* → ILC3 → serum amyloid A1 and A2 → the development of Th17 cells	-
	Clostridium → Treg cell differentiation → suppresses immune responses	Yang et al. (2020)
	*Bifidobacteria* → dendritic cells → Th1 cells → IFN-γ → suppression of Th2 cells → R suppression	Li et al. (2000); Krueger et al. (2021)

The tables summarize major microbial and metabolite alterations in AR patients along with their core mechanisms. (1) (A) Outlines changes in lung and intestinal microbial structures in AR patients. (2) (B) Summarizes alterations in metabolite levels within AR patients. (3) (C) Details the mechanisms underlying the metabolite changes in Table B. The following is an explanation of each item in the rightmost column of C, starting from the top: (a) *Staphylococcus aureus* lysate promotes IgE secretion to aggravate AR. (b) *Streptococcus salivarius* promotes the expression of IL-6, IL-8, TNF-α, and CCL11, which chemotaxis eosinophils and causes them to secrete pro-inflammatory factors and then aggravate AR. (c) *Staphylococcus aureus* promotes ILC3 to produce IL-22. IL-22 promotes the production of serum amyloid A1 and A2, and then the latter promotes the development of Th17 cells to aggravate AR. (d) *Clostridium* suppresses immune responses by promoting Treg cell differentiation through IL-10 secretion from intestinal epithelial cells. (e) Cell wall peptidoglycan of *Bifidobacteria* is recognized by Toll-like receptors (TLRs) on the surface of immune cells, such as dendritic cells, prompting the dendritic cells to secrete IL-12. Subsequently, IL-12 promotes the differentiation of naive T cells into Th1 cells and stimulates these Th1 cells to secrete gamma interferon (IFN-γ). IFN-γ ultimately directly inhibits the proliferation and function of Th2 cells, suppressing their ability to secrete pro-inflammatory cytokines, thereby alleviating AR.

## 4 Limitations of microbial regulation of the lung and intestines

Regulatory therapies for the pulmonary intestinal microbiota are not without limitations.

Individual differences in patients will directly affect the effectiveness of treatment. Patients with underlying disruptions in their pulmonary or intestinal microbiota inherently possess a weaker capacity for inflammation suppression mediated by these microorganisms. Consequently, antibiotic use simultaneously devastates beneficial bacterial populations within their bodies, leading to prolonged inability to recover. Therefore, the best treatment plan for these patients is to increase the species and number of beneficial bacteria to inhibit the harmful bacteria through competition between the microbiota. However, a significant limitation of probiotic and FMT approaches is their variable and often delayed efficacy, which may not suffice for patients requiring immediate symptom relief.

Stability of colonization is also an essential factor in efficacy. The exploration of a bacterial treatment that can automatically maintain a stable level of colonization with a small amount of bacteria over a considerable length of time is the desired effect. And many probiotic treatments have difficulty in meeting this characteristic and need to be taken over a considerable length of time, which also greatly increases the financial cost to the patient.

Safety is also one of the factors we should consider. Many antibiotics can easily kill harmful bacteria, but at the same time, beneficial bacteria suffer loss, which is not the result that doctors and patients want to see when treating diseases at the expense of their own health status. Furthermore, whether the destroyed probiotic microbiota can be automatically maintained at a stable health level through colonization is still a question. This is because we do not know exactly what probiotic colonies are destroyed, and secondly, whether these destroyed colonies have a symbiotic or antagonistic effect, as well as the relationship between the colonies is not yet clear. If patients need long-term medication after receiving the treatment, it will be a huge financial burden for them.

Interestingly, the academic community initially held divergent views on whether microbial changes were the cause or consequence of AR inflammation. However, a substantial body of existing research predominantly supports the notion that microbes are a cause of AR inflammation. For instance, a human study utilizing *in vitro* and *in vivo* AR models demonstrated that *Streptococcus salivarius* contributes to AR development by promoting the release of inflammatory cytokines and inducing morphological changes in nasal epithelial cells (Miao et al., 2023). The reason for this debate likely stems from the limited early research on the mechanisms of AR, which primarily focused on the correlation between AR and changes in the human microbiome. It was precisely these studies on the association between AR and microbiome alterations that prompted researchers to further explore whether these microbial changes are the cause or the consequence of AR inflammation.

## 5 Intestinal microorganisms influence AR via the intestinal-pulmonary axis

Numerous contemporary studies have revealed that intestinal microbiota can mitigate the advancement of pulmonary diseases by boosting respiratory immunity and eliminating pathogens via microbial metabolites. Conversely, pulmonary disorders can alter the structural integrity and microbial diversity of the intestinal microbiota, leading to associated gastrointestinal symptoms (Zhou et al., 2020). Contemporary medical science designates the bidirectional communication network between the gastrointestinal and respiratory systems as the “intestinal-lung axis” (Budden et al., 2017). This intestinal-lung axis network primarily refers to the bidirectional regulation between intestinal and respiratory tract microbiota through multiple pathways, including immune factors and microbial metabolites circulating in the blood and lymphatic systems (Deepika Alsharari et al., 2025). It has advanced our comprehension of the correlation between intestinal microbiome composition and AR. The nose is connected to the lungs and belongs to the same respiratory system, so AR is categorized as a lung disease. Below, we will explain how intestinal microbiota specifically influence AR through the intestinal-lung axis.

The specific mechanism by which intestinal microbiota influence AR pathogenesis through the intestinal-lung axis.

### 5.1 Immunomodulatory effects

The intestinal microbiota maintains immune system tolerance and reduces allergic reactions by promoting the production or secretion of regulatory T cells (Tregs). For example, *Bacteroides* can secrete polysaccharides that induce Tregs to secrete IL-10 (Neff et al., 2016). And IL-10 directly suppresses the activation and proliferation of Th2 cells, thereby reducing the secretion of proinflammatory cytokines such as IL-4 and IL-5, which alleviates allergic rhinitis. The primary physiological Role of Treg cells is to directly contact or secrete cytokines with immunosuppressive effects, with representative examples being TGF-βand IL-10 to exert immunosuppressive effects on diverse immune cells. (Treg cells themselves can play an immunosuppressive role by contacting these factors, and at the same time, they can secrete these factors to act on other immunosuppressive cells). Also, as mentioned above, the commensal bacterium *Clostridium* promotes the differentiation of Treg cells through the secretion of IL-10 from intestinal epithelial cells, and the proliferation and differentiation of Treg cells can secrete more anti-inflammatory factors, thereby suppressing immune responses (Yang et al., 2020).

### 5.2 Maintenance of intestinal barrier function to alleviate AR

The commensal intestinal microbiota preserves intestinal epithelial barrier function, thereby inhibiting translocation of pathogens and noxious compounds, and reducing systemic inflammatory responses (including localized inflammatory responses in the nose). As mentioned above, *Bifidobacteria* can also activate plasma cells under the intestinal epithelium to produce secretory immunoglobulin IgA, which protects the body and suppresses inflammation. This suggests that when this barrier is disrupted, it can be treated with the help of targeted colonization by *Bifidobacteria*.

Early lymphoid tissue-inducing cell (LTi) [This cell is a group of prenatal lymphoid tissue-inducing cells, characterized by high chemokine receptor 6 (CCR6) expression, which is important for the formation of lymphoid tissues such as lymph nodes] formation appears to be microbiota-independent, as germ-free (GF) mice exhibit normal development of lymph nodes and Peyer’s patches, suggesting commensal bacteria are dispensable for lymphoid tissue organogenesis. (Pyle’s nodes are a group of lymphoid follicles within the small intestinal mucosa). The surface of Pyle’s node is covered with a layer of microscopic wrinkled cells, also known as M cells. It recognizes many antigens presented in the gastrointestinal tract and mainly phagocytizes viruses and enteropathogenic bacteria, delivering the swallowed antigens in the intestinal lumen to the immune cells, which can process, transit, and present the pathogenic antigens. In this process the activated immune cells go through the circulatory system and return again to the intestinal mucosa lamina propria to become secretory IgA (an immunoglobulin that neutralizes pathogens and also regulates the strength and direction of the immune response to avoid excessive inflammatory reactions that can cause harm to the body.) Plasma cells and effector T cells, which are predominantly plasma cells, participates in the intestinal mucosal immunity (Mabbott et al., 2013). Since the circulatory system also connects to the respiratory system, we hypothesize that immune cells activated in the intestine during the aforementioned process can also reach the nasal cavity via the circulatory system to regulate inflammatory responses. This may represent a novel breakthrough in elucidating the intestinal-lung axis.

### 5.3 Intestinal epithelial cells

**Figure 1:**
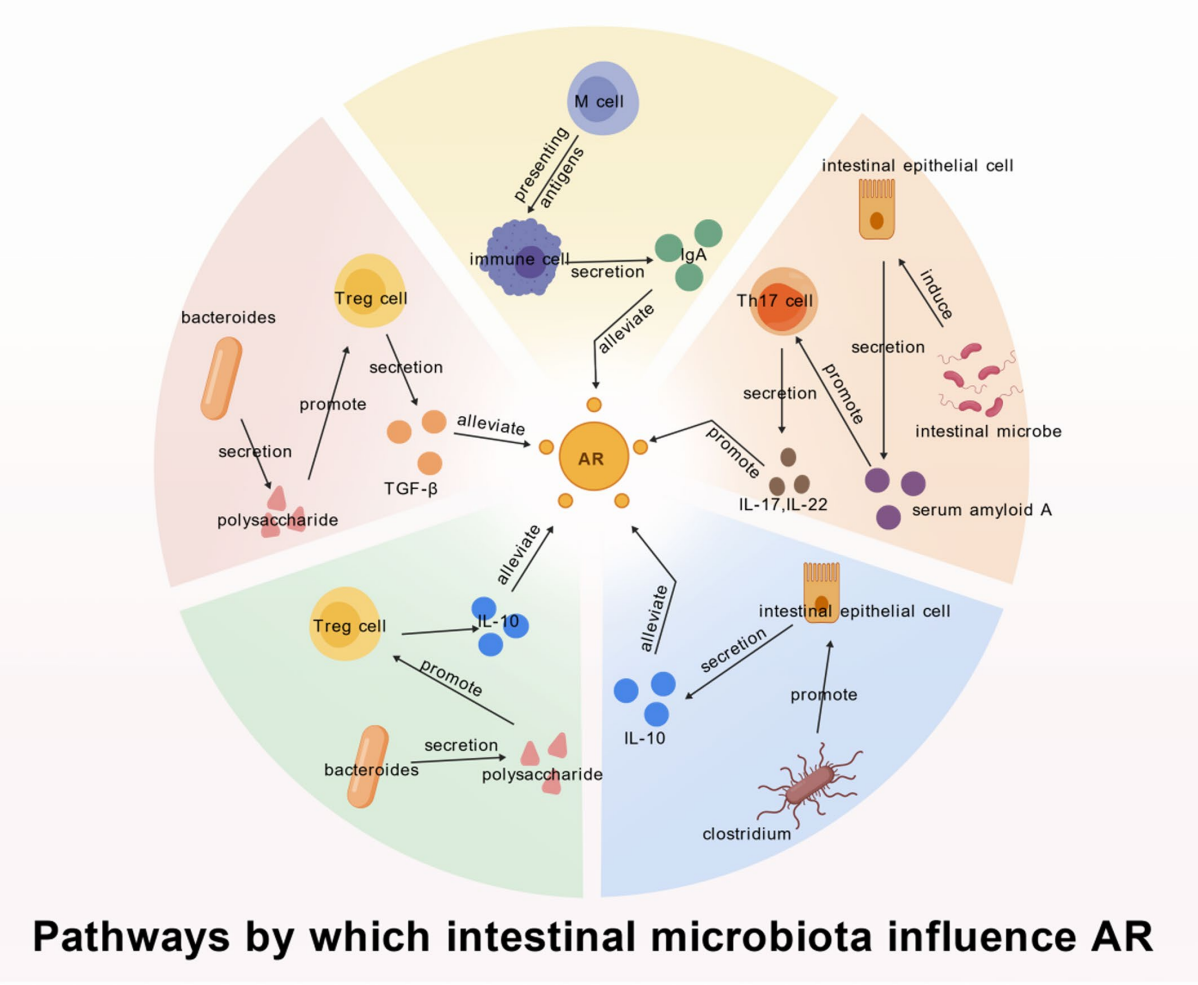
Pathways by which intestinal microbiota influence AR. (1) The red fan-shaped represents that *Bacteroides* can secrete polysaccharides that promote Treg differentiation. Treg cells chiefly function to directly contact or secrete cytokines with immunosuppressive effects, including TGF-βto exert immunosuppressive effects on a variety of immune cells, thus alleviating AR (Neff et al., 2016). (2) The green fan-shaped represents that *Bacteroides* can secrete polysaccharides that promote Treg differentiation, then secrete IL-10 to alleviate AR. And IL-10 directly suppresses the activation and proliferation of Th2 cells, thereby reducing the secretion of pro-inflammatory cytokines such as IL-4 and IL-5, which alleviates allergic rhinitis (Neff et al., 2016). (3) The blue fan-shaped represents that the commensal bacterium *Clostridium* suppresses immune responses by promoting Treg cell differentiation through IL-10 secretion from intestinal epithelial cells (Yang et al., 2020). (4) The orange fan-shaped represents that Microorganisms colonize the intestine and induce serum amyloid A production via intestinal epithelial cells, which improves Th17 differentiation and produces IL-17 and IL-22, thereby inhibiting AR (Teruyuki et al., 2015). (5) The yellow fan-shaped represents that M cells can recognize many antigens presented in the digestive tract, delivering swallowed intestinal lumen antigens to the immune cells, immune cells can be processed into pathogenic antigens, transit, and delivery. In this process, the activated immune cells go back to the intestinal mucosal lamina propria through the circulatory system, and become plasma cells and effector T cells mainly secreting IgA to participate in the intestinal local immune response. IgA is a kind of immunoglobulin, which can neutralize pathogens, and can also regulate the intensity and direction of immune response to avoid excessive inflammatory response in the body to cause harm (Mabbott et al., 2013).

Intestinal epithelial cells are the immune system that connects the bacteria to the host. They decode signals from symbiotic bacteria (including metabolites, bacterial components, and whole bacteria) and relay them to mucosal immune cells (Agnieszka et al., 2022). In addition, microorganisms colonize the intestine and induce serum amyloid A production via intestinal epithelial cells, which improves the differentiation of Th17 (Th17 cells mainly secrete cytokines such as IL-21, IL-17, and IL-22, which stimulate the production of pro-inflammatory factors by a variety of cells and recruit neutrophils to initiate inflammatory responses) and the production of IL-22 (Teruyuki et al., 2015). This immunomodulatory pathway within the intestinal-lung axis is summarized in Figure 1. Therefore, intestinal epithelial cells can be considered the “mediator” through which intestinal microbiota influence nasal inflammation. A systematic review on interleukin-22 in allergic airway diseases indicates that IL-22 levels correlate positively with total immunoglobulin E (IgE), specific IgE, and eosinophil counts in the nasal mucosa (Tamasauskiene and Sitkauskiene, 2020). We therefore hypothesize that it promotes inflammation by stimulating IL-22 release, which then acts via the blood-lymphatic circulation of the intestinal-lung axis on IgE-releasing B lymphocytes in the nasal mucosa.

### 5.4 Key immune pathways in the intestinal-lung Axis

Short-chain fatty acids (SCFAs), the primary metabolites produced by intestinal microbiota during dietary fiber breakdown, promote the differentiation of naive T lymphocytes into Treg cells within the intestine (Kaisar et al., 2017). The promotion of Treg cell differentiation increases their numbers, leading to higher levels of anti-inflammatory factors such as IL-10 and TGF-β secreted at the same concentration. Consequently, AR can be remotely regulated through the blood system of the intestinal-lung axis, thereby alleviating AR symptoms.

A mouse model experiments have demonstrated that an imbalance between helper T cells 17 (Th17) and Tregs is closely associated with the pathogenesis of AR (Van Nguyen et al., 2020). The balance between Th17 and Tregs can serve as a complementary concept to the intestinal-lung axis. Both Tregs and Th17 cells originate from CD4 + T cells and are mediated by a common signaling pathway involving transforming growth factor-β (TGF-β). Th17 cells secrete the proinflammatory cytokine IL-17, which induces specific inflammatory responses in the nasal cavity. A clinical study conducted on humans indicates that IL-17 correlates with the severity of clinical symptoms and inflammation (Bayrak et al., 2018). Under Treg-inducing conditions, T cells fail to differentiate into Th17 cells and ultimately develop into Foxp3 + Treg cells. These Treg cells subsequently secrete IL-10 to suppress AR. Notably, *Clostridium* species belonging to clusters IV and XIVa in the intestine enhance the accumulation of Treg cells (Atarashi et al., 2011). This indicates that *Clostridium* species clusters IV and XIVa may indirectly suppress AR. Furthermore, the colonization of *Clostridium* in the intestine creates a rich TGF-β environment, which influences both intestinal and systemic immune states—including those associated with AR—by increasing the number of Foxp3 + Treg cells (Atarashi et al., 2013).

Tryptophan, as a key intestinal metabolite, undergoes metabolism via three distinct pathways under steady-state conditions. The primary pathway is the kynurenine pathway (KP), catalyzed by indoleamine 2,3-dioxygenase 1 (IDO1). Second, pathways promoting allergic rhinitis: serotonin production within intestinal chromaffin cells, a process catalyzed by tryptophan hydroxylase 1 (TpH1); Third, the direct conversion of tryptophan into indole and its derivatives, as well as tryptamine, via tryptophanase through the intestinal microbiota, leading to the synthesis of multiple metabolites. Research indicates that the first pathway (kynurenine pathway) enhances Foxp3 transcription factor expression, a process that promotes Treg cell differentiation while suppressing Th17 cell proliferation, thereby inhibiting AR. The second pathway, however, converts Treg cells into Th17 cells through dendritic cells (DCs), further triggering or promoting AR (Yang et al., 2022). The third pathway involves reducing T-cell responsiveness and stimulating Treg production through various indole molecules formed by the intestinal microbiota’s catabolic metabolism of tryptophan, thereby alleviating AR. Notably, *Clostridium* species such as *Clostridium botulinum*, *Clostridium thermophile*, and *Clostridium sporogenes* can convert tryptophan into indolepropionic acid (IPA) (Yang and Cong, 2021). IPA promotes the differentiation of regulatory Treg cells and suppresses Th2 immune responses. This mechanism alleviates AR by increasing the release of anti-inflammatory factors from Treg cells and decreasing the release of pro-inflammatory Th2 factors.

The preceding three paragraphs describe the key pathways through which the intestinal-lung axis influences nasal target organs via immune molecular mechanisms, thereby affecting AR.

## 6 Hypotheses regarding the intestinal-lung axis mechanism

In terms of the systematic practice of traditional Chinese medicine, the lungs and large intestine each have their own meridians, and the lungs and large intestine are connected through these meridians. In traditional Chinese medicine, the physiology and pathology of the lungs and large intestine can influence each other, which provides us with ideas and possibilities for exploring the lung-intestine axis. However, traditional Chinese medicine theory is based solely on practice and does not have a theoretical foundation or references in reality.

The intestinal-lung axis is the pathway by which intestinal microbiota affects the immune response in the lungs through blood, lymphatic, and neural pathways (Deepika Alsharari et al., 2025). The intestinal-lung axis is a mechanism that provides novel therapeutic avenues for AR. Through an in-depth understanding of the intestinal-lung axis, we have conducted innovative and thorough investigations into its specific mechanisms. We propose that the intestinal-lung axis may also function as a cellular pathway—specifically, basophils migrating via the blood-lymphatic system to the lungs to exert an inhibitory effect on AR. For example, An animal study indicates that compared to mice with allergic rhinitis, those orally administered *Enterococcus faecalis* (LFK) had a significant reduction in eosinophils infiltrating the nasal mucosa. This indicates that intestinal microbiota can influence the influx of eosinophils into the nasal cavity of mice (Zhu et al., 2012). So we hypothesize that alterations in intestinal microbiota composition may similarly reduce the influx of basophils into the nasal cavity of mice.

Our hypothesis and discussion are as follows. First, interactions between the intestinal and lung microbiomes may either enhance or mitigate allergic reactions by jointly regulating immune responses. And the bridge facilitating communication between the intestinal-lung axis is likely another cell type, basophils. The interplay between intestinal and lung microbiota may enhance or slow allergic responses by co-regulating immune responses, and the bridge between the intestinal-lung axis of intercommunication is likely to be basophils. For example, a clinical studies have shown that children who suffer from atopic dermatitis in the early stage are prone to food allergy and asthma in the later stage (Brough et al., 2015). Based on existing evidence, we hypothesize and infer that the likely cause is impaired early skin surface barrier function. When stimulated by certain external irritants, epithelial cells produce IL-25 and IL-33, activating type 2 innate lymphoid cells and other immune cells to secrete type 2 cytokines IL-4, IL-5, and IL-13. This promotes Th2 cell development and eosinophil recruitment. Th2 cell-dependent B cell activation promotes the production of allergen-specific IgE, which subsequently binds to basophils (Eggel et al., 2024). When the same external stimuli are inhaled into the lungs, type I alveolar cells, possessing endocytic and exocytic functions, sequentially transport these stimuli from the alveolar inner wall (exposed to the external environment) to the pulmonary interstitium via endocytosis and exocytosis. Macrophages then acquire these stimuli and secrete inflammatory mediators. These inflammatory mediators activate vascular endothelial cells to express the adhesion molecule VCAM-1, facilitating leukocyte migration from blood vessels into inflammatory sites (Singh et al., 2023). Given that basophils belong to the leukocyte family, we hypothesize that basophils may be recruited to the lungs via this chemotactic pathway. This hypothesis explains why IgE-coated basophils, upon re-exposure to the same external stimulus, trigger allergic reactions at the corresponding site. It also accounts for the phenomenon where children with early-onset atopic dermatitis later develop food allergies and asthma—conditions occurring in distant sites yet exhibiting a synergistic effect. Furthermore, the fact that IgE can remain bound to basophils on the cell surface for months or even years provides another theoretical explanation for the time lag observed between the onset of atopic dermatitis and the development of subsequent food allergies and asthma.

Therefore, investigating whether basophils serve as the axis connecting the lungs and the intestine could significantly aid future diagnostics in combating the organ-to-organ transfer of allergic diseases. However, this theory requires substantial research to validate. Whether basophils in the intestine can replicate the chemotactic mechanisms of leukocytes remains to be investigated. We propose an experimental approach: labeling isolated basophils using fluorescent markers, then injecting them into the intestines of mice with AR. Chemically stimulating the intestinal microbiota to release pro-inflammatory factors. Validation would be achieved by detecting the presence of fluorescently labeled basophils in the pulmonary interstitium of these mice using fluorescence detection techniques. The detection of fluorescently labeled basophils in the lungs or pulmonary interstitium would provide preliminary confirmation of this hypothesis.

By comparing the effects of lung and large intestine bacteria on AR, we found that *Staphylococcus aureus* is present in both the large intestine and lungs and can exacerbate AR. Based on relevant literature, we made another hypothesis to explain the intestinal-lung axis: certain signal molecules exist within certain bacterial communities, allowing bacteria to sense each other and trigger related reactions in other areas. Meanwhile, some literature (University of Science and Technology of China, 2010) suggests that during proliferation, bacteria produce chemical signals that transmit information between bacterial cells. The concentration of these signals accumulates extracellularly as bacteria proliferate. When these concentrations reach a certain threshold, they can be detected by bacteria, which then bind to corresponding receptors, triggering a series of gene regulatory responses across the population. This enables bacteria to coordinate their actions at the multicellular level to perform important physiological functions. The experiment also revealed that the AI-2 quorum-sensing system regulates the expression of capsular polysaccharides through the binary signaling system KdpDE, thereby influencing phenotypic changes and controlling biofilm formation and bacterial resistance to phagocytosis. This literature demonstrates that chemical signals produced by bacteria can be regarded as instructions. When the concentration of these signals reaches a certain threshold, other bacteria collectively execute related gene regulation and expression, thereby completing a reaction that influences one site from another. This also indirectly supports our hypothesis. However, this evidence remains insufficient because the exploration of these signaling molecules is incomplete, and it remains unknown whether they can achieve remote regulation from the colon to the nose or lungs via the circulatory or lymphatic systems. For example: Can AI-2 produced by intestinal bacteria remotely regulate the KdpDE system in corresponding bacteria within the lungs and nose? Could this enhance the anti-phagocytic properties of these microbial communities, leading to dysbiotic proliferation and exacerbating allergic rhinitis? These problems require further investigation.

There are also studies actively exploring the mechanisms underlying the intestinal-lung axis, such as Tianyi et al. (2024) the altered intestinal microbiota induced by polysaccharides (POP) effectively alleviated type 2 inflammation, reduced the number of ILC2 cells in the lungs and intestines, and inhibited the migration of intestinal ILC2 cells to the lungs, thereby providing some relief for AR. However, these findings are still insufficient to clearly elucidate the specific mechanisms of the intestinal-lung axis. Perhaps the intestinal-lung axis connects the lungs and intestinal microbiota through multiple pathways and exerts a complex, cross-influencing effect on AR.

## 7 Metabolites

Metabolites are intermediate or final products produced by a series of biochemical reactions in the metabolic process of an organism. In the course of studying microorganisms, the researchers found that microbial metabolites, especially short-chain fatty acids and arachidonic acid, have significant changes on the human body. And these metabolites, in turn, have the potential to be used as markers for the future diagnosis of AR, which will provide a more diagnostic basis for future clinical diagnosis.

### 7.1 Short-chain fatty acids (SCFA)

Short-chain fatty acids are volatile organic acids containing fewer than six carbon atoms, primarily comprising acetate, propionate, and butyrate (Saha et al., 2025). Their origin is closely related to the metabolic activity of probiotic and anaerobic bacteria in the intestinal tract, the main source being their fermentation of dietary fiber, resistant starch, and oligosaccharides. It has a significant anti-inflammatory effect and can inhibit the release of intestinal inflammatory factors, thus inhibiting AR. For example, a study demonstrates that SCFA produced by microorganisms promotes the production of anti-inflammatory mediators IL-10 by macrophages (Liu et al., 2012; Wen et al., 2021). And IL-10 inhibits the production of pro-inflammatory cytokines by macrophages and monocytes, thereby reducing the inflammatory response. In addition, Microbiota-derived short-chain fatty acids (especially butyric acid) generated through dietary fiber fermentation inhibited ILC2 (type II intrinsic lymphoid cells) function and prevented lung inflammation (Lewis et al., 2019).

Despite this, there are still studies that seem to contradict these results. Related studies have shown that SCFA altered macrophage metabolism, decreased mTOR kinase activity, and increased the production of anti-microbial peptides (Schulthess et al., 2019). Antimicrobial peptides stimulate innate immune cells to augment pathogen clearance capacity, consequently exacerbating inflammatory responses in allergic rhinitis.

Propionic acid and butyric acid have been reported to significantly inhibit histone deacetylase, and butyric acid has the strongest inhibitory activity (Maslowski Kendle et al., 2009). And histone deacetylase is involved in the expression of IL-10 to suppress inflammation. Whereas acetic acid maintains the balance of the immune system and suppresses excessive inflammatory responses, this explains the different effects of different fractions of SCFAs on AR. This may also explain the contradictory results described above. In addition, acetic acid can also be used as a substrate to be converted into butyric acid by bacteria such as *Firmicutes* (Zhao et al., 2025). Does this indirectly suggest that we can modulate the isoforms of short-chain fatty acids to play different roles by injecting them into bacteria such as *Firmicutes* according to different needs during the treatment of AR? It is worth noting that butyric acid itself also has anti-inflammatory and anti-allergic effects, but butyric acid can also stimulate a more intense inflammatory response, suggesting that how to control butyric acid in a targeted manner to produce the intended effect is still a question.

Furthermore, dendritic cells (DCs) serve as pivotal orchestrators of adaptive immunity. They infiltrate into secondary lymphoid tissues and stimulate CD4 + T cells to exhibit subset diversification grounded in activation signals. Symbiotic bacteria-derived metabolites affect the function of DCs. For example, the secretion of IL-12 is inhibited by SCFAs, but IL-10 and IL-23 are upregulated (Xie et al., 2025). IL-10 inhibits T cell activation and proliferation and suppresses dendritic cell maturation, whereas immature dendritic cells induce immune tolerance. Because in the immature stage, although these dendritic cells are able to efficiently capture and process antigens, they are relatively weak in their capability to process and present antigens to T cells. And dendritic cells exhibit low surface expression of co-stimulatory molecules, which limits their T cell-activating potential to mitigate allergic reactions. IL-23 drives Th17 cell to secrete IL-17 and IL-22, amplifying the local inflammatory response. Another study showed that microbial-derived SCFAs induce dendritic cell process elongation toward the intestinal lumen by inhibiting an enzyme known as histone deacetylase, causing them to express more cell surface co-stimulatory factors after maturation and increasing the rate of antigen presentation, which leads to stronger immune responses (Takuho et al., 2023). This conclusion is ostensibly in complete contradiction to the previous one. In contrast to the above literature, which states that SCFA inhibits dendritic cell maturation by increasing IL-10 production, the following study concludes that SCFA triggers dendritic cell protrusion and maturation through a different pathway—inhibition of histone deacetylase. Why do SCFAs enhance the secretion of pro-inflammatory factors while suppressing inflammation? Through literature review and analysis, we discovered a specific type of SCFA—branched-chain short-chain fatty acids (BCSFA)—produced by *Clostridium* fermentation of proteins. It can promote inflammation, though the mechanism remains unclear. Furthermore, no studies have demonstrated that it is unrelated to AR; rather, it is more closely associated with other diseases. However, by observing the pro-inflammatory effects of branched-chain short-chain fatty acids in other diseases, we analogize to topics related to allergic rhinitis. The most likely reason is that branched-chain short-chain fatty acids may act as signaling molecules to recruit and activate innate immune cells such as neutrophils, thereby triggering or exacerbating local inflammatory responses. We hypothesize another possibility: even if the above mechanism exists, patients with insufficient protein intake may produce minimal SCFAs. Combined with the relatively weak pro-inflammatory effect of IL-23 due to individual variability, sometimes insufficient to produce a significant pro-inflammatory effect. Consequently, this mechanism may have gone unobserved in previous experiments, leading researchers to mistakenly conclude that SCFAs solely exert an inhibitory effect on inflammation, while data regarding IL-22’s pro-inflammatory role were overlooked.

### 7.2 Arachidonic acid (ARA)

Arachidonic acid (ARA) is widely found in nature and is one of the important lipids in living organisms. It is found in the human body mainly in the phospholipids of cell membranes. Metabolomic analyses indicate significant changes in metabolites related to ARA metabolism in the serum of AR patients, such as prostaglandin D2 in the arachidonic acid metabolic network (Yuan et al., 2022). It suggests that the initiation and evolution of AR may be related to abnormalities in ARA metabolism. While the main sources of ARA include animal foods (such as animal liver, some nuts, and breast milk), this suggests that arachidonic acid is closely related to food allergies, and suggests that AR can be treated indirectly by dietary control, such as reducing the consumption of the above foods.

Our results demonstrate markedly elevated prostaglandin D2 concentrations in allergic rhinitis patients compared to healthy controls (Yuan et al., 2022). Whereas (PG) D2 is produced from arachidonic acid via the cyclooxygenase pathway. Cyclooxygenase is the primary enzyme that facilitates the conversion of arachidonic acid to prostaglandins, and it has been found that cyclooxygenase has two isozymes, COX-1 and COX-2 (Dong and Malkowski, 2025). The former of which is an inducible enzyme, and multiple noxious stimuli (chemical, physical, and biological) induce phospholipase A2 activation, which cleaves membrane phospholipids to release arachidonic acid (ARA), which is produced through COX-2 catalyzed oxygenation to produce prostaglandins. In addition, mast cells and Th2 cells are likely to mediate epithelial barrier dysfunction in AR, causing their destruction (Steelant et al., 2017). This may suggest that arachidonic acid is produced when the cell membranes of the cells that make up the epithelial barrier are broken down or disrupted.

PGD2 may serve as a valuable biomarker since IgE-mediated mast cell degranulation releases PGD2, subsequently stimulating eosinophil and basophil activation (Yuan et al., 2022), and causes them to degranulate to release pro-inflammatory factors, thereby triggering allergic reactions.

### 7.3 The role of tryptophan in AR

**Figure 2:**
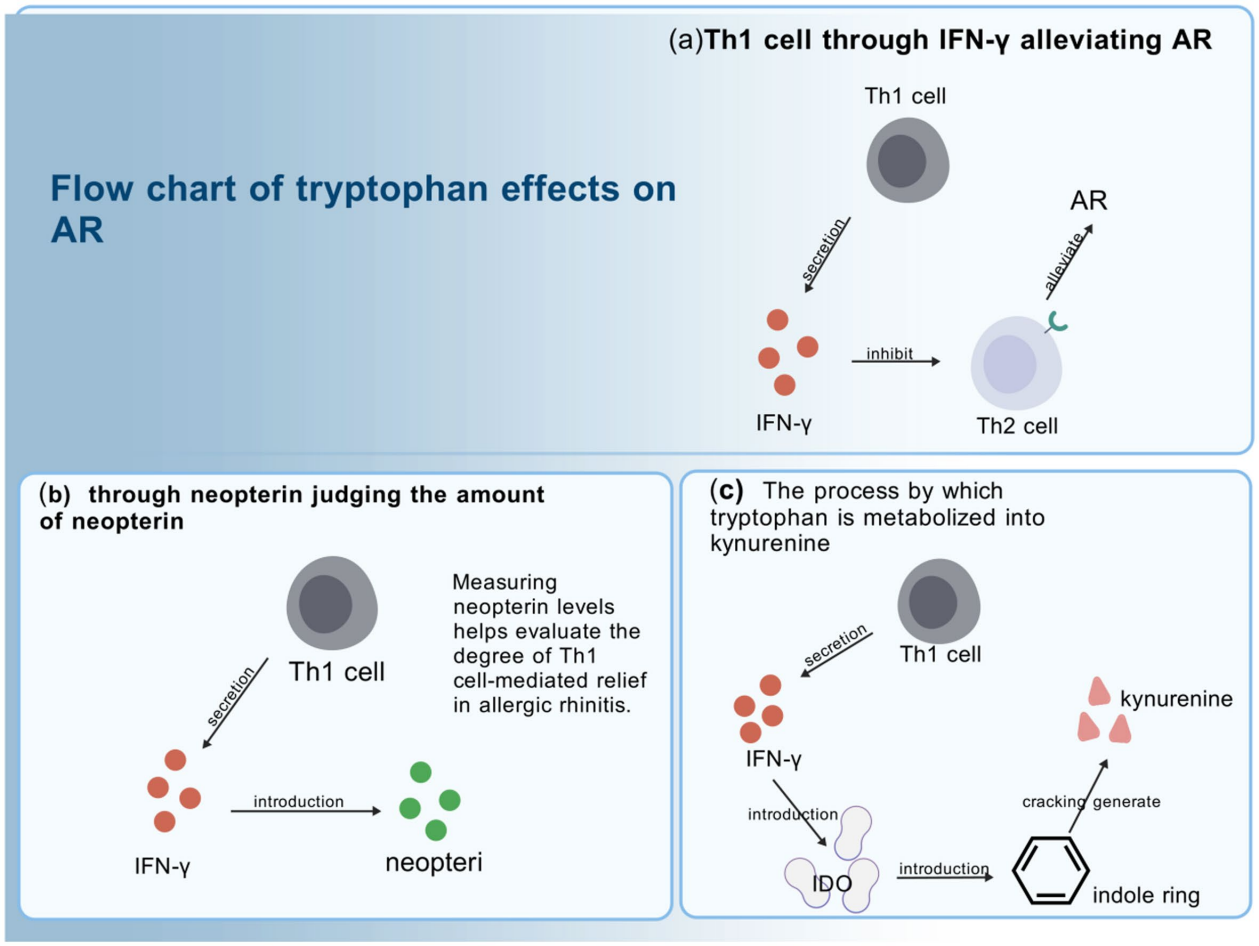
Flow chart of tryptophan effects on AR. **(a)** The cytokine IFN-γ produced by Th1 cells, can induce both the gene expression and enzymatic activity of IDO. While inducing IDO, IFN-γ also suppresses the development and function of Th2 cells, thereby alleviating AR. **(b)** The cytokine IFN-γ secreted by Th1 cells, can stimulate neopterin production. Since IFN-γ also promotes the occurrence of pathway **(c)**, quantitative analysis of neopterin levels can reflect the extent of tryptophan conversion into kynurenine, indirectly indicating the degree of IFN-γ-mediated alleviation of AR. **(c)** The cytokine IFN-γ from Th1 cells induces the gene expression and enzymatic activity of indoleamine 2,3-dioxygenase (IDO). The kynurenine pathway of tryptophan metabolism begins with the cleavage of the indole ring of tryptophan, a process in which IDO acts as a catalyst.

Tryptophan, an essential amino acid, undergoes bifurcated metabolism through three principal routes: serotonin synthesis, indole pathway, and kynurenine generation (Xue et al., 2023). Among these, the kynurenine metabolic pathway begins with cracking of the indole ring on tryptophan, and there are two principal enzymes governing tryptophan catabolism—hepatic tryptophan 2,3-dioxygenase (TDO) and widely expressed indoleamine 2,3-dioxygenase (IDO), which is derived from macrophages, microglia, or dendritic cells (Yiquan and Guillemin Gilles, 2009). One of the cytokines of Th1 cells, IFN-γ, stimulates IDO gene transcription and enhances its catalytic function. Tryptophan is converted to kynurenine under the induction of the TDO/IDO enzyme. And IFN-γ induces IDO while suppressing Th2 cell differentiation and effector functions, which is important for preventing excessive allergic reactions. IFN-γ also induces neopterin production. Thus, a decrease in tryptophan content, as well as an increase in kynurenine and neopterin, can be used as a marker for remission of AR.(see Figure 2 for the flow of tryptophan in relation to AR).

A human clinical trial has demonstrated that pollen-sensitive AR patients demonstrate peak symptom severity in spring months when pollen is more abundant and tryptophan degradation is reduced, so that kynurenine and neopterin levels are reduced (Giorgio, 2010). This suggests that the suppression of the function of Th1 cells to inhibit inflammation in patients with pollen-induced AR results in the secretion of less IFN-γ, which therefore leads to a reduced degree of tryptophan degradation and lower levels of kynurenine and neopterin. This suggests that kynurenine and neopterin can be used as biomarkers for pollen-induced AR for future clinical diagnosis.

## 8 Treatment of AR

The rising incidence of AR and the soaring cost of treatment have led researchers to increase their search for therapies with immunomodulatory potential. First-line AR management relies on nasal corticosteroid anti-inflammatory effects complemented by H1 receptor blockade through antihistaminic agents (Siddiqui et al., 2022). The former can reduce the inflammation of the nasal mucosa, alleviate nasal obstruction, rhinorrhea, sneezing paroxysms, and associated symptomatology, but cannot be completely cured. The latter can inhibit mast cell degranulation, which in turn reduces the release of histamine. Anticholinergic drugs are also used, which can lead to a decrease in cholinergic nerve activity. This leads to a reduction in nasal secretions to achieve relief of symptoms such as a runny nose, and anti-leukotrienes can reduce leukotriene-induced inflammation and airway constriction, which can be effective in AR. However, the effectiveness of these drugs is limited, and they are accompanied by a variety of side effects (Bousquet et al., 2020). Therefore, it has become necessary to open new therapeutic pathways in the treatment of AR. Currently available novel treatment approaches primarily fall into two categories: (1) Fecal Microbial Transplantation (FMT); (2) Bacterial Lysate Therapy. Meanwhile, established standardized therapies encompass four categories: (1) Probiotics; (2) Dietary Therapy; (3) Prebiotics; (4) Antibiotic Therapy.

### 8.1 Emerging therapies

#### 8.1.1 Potential for microbiota transplantation

Restoring the normal microbial community through intestinal or lung microbiota transplantation may be helpful in relieving the symptoms of AR. Fecal microbiota transplantation (FMT), a new therapy, may ensure the maintenance of a stable intestinal microbiota. FMT is a therapeutic procedure involving the preparation of donor-derived fecal material into a processed suspension for intestinal delivery, aiming to restore intestinal microbial homeostasis (Roni et al., 2019). For example, relevant literature describes that FMT can treat inflammatory bowel disease by remodeling the intestinal microbiota (Ooijevaar Rogier et al., 2019). While no clinical trials have yet investigated FMT for AR management, this modality holds significant promise for intestinal microbiome restoration in AR.

FMT may demonstrate superior efficacy to oral probiotics by delivering orders of magnitude greater microbial diversity and enabling stable engraftment of commensal communities (Grehan et al., 2010). This hypothesis finds support in the clinical trial conducted by Mashiah et al., which demonstrated FMT’s immunomodulatory effects in atopic dermatitis patients. The study demonstrated a significant reduction in pruritus scores after FMT. In addition, the number of weekly topical corticosteroid applications was reduced during both the active treatment and subsequent monitoring periods. Macrogenomic analysis of the intestinal microbiota showed significant transmission of bacterial strains from donor to patient. No adverse effects were observed with treatment (Jacob and Tal, 2022). Notwithstanding, current evidence remains inadequate to substantiate its therapeutic efficacy in AR, necessitating large-scale randomized controlled trials for definitive conclusions.

#### 8.1.2 Bacterial lysates

Bacterial lysates (BLs) are immunomodulators made up of antigens from respiratory pathogens. The most common include grass green Streptococcus, Catamorium, *Streptococcus pyogenes*, *Haemophilus influenzae* (Kaczynska et al., 2022). BLs may be delivered via oral, intranasal, or sublingual routes. The therapeutic mechanism of BLs relies on simulated pathogen antigen exposure to induce immunological reactions. They promote the release of antiviral cytokines, activation of NK cells, and restoration of the Th1/Th2 balance by activating DCs through Toll-like receptors. In-depth mechanisms of BLs’ action refer to Kaczynska et al. (2022).

A study performed an open-label sequential investigation examining OM-85’s impact on respiratory infection frequency, exacerbations of primary disease, and the severity of symptoms in patients with AR. Patients underwent three treatment cycles, each featuring a 10-day therapeutic course and a subsequent 20-day medication-free interval. They found that The study demonstrated that OM-85 treatment significantly decreased both respiratory infection frequency and AR exacerbation rates, while also attenuating allergy symptom severity relative to baseline optimized standard care. Furthermore, the study revealed elevated IgA concentrations in both serum and salivary samples (Koatz et al., 2016). Another study with the same way observed that nasal IFN-γ (IFN-γ promotes Th1-type immune responses by inhibiting the development of Th2 cells, which is important for preventing AR.) Elevated levels, decreased levels of nasal IL-4 and IL-13 (IL-4, IL-5 are both pro-inflammatory cytokines; Meng et al., 2019).

In summary, diet, prebiotics, probiotics, antibiotics, microbiota transplants, and bacterial lysates have all been shown to have a good alleviating effect in AR. However, these aspects are not widely used in the clinic at present, and their specific usage and mechanisms still need to be explored in more clinical trials.

### 8.2 Standard treatment for AR

#### 8.2.1 Probiotics

Probiotics are viable microbial strains that undergo stringent selection based on demonstrated health-promoting effects when administered at adequate doses (Gregor et al., 2019).” Probiotics, a new treatment for AR, are able to reduce nasal allergy symptoms by regulating the composition of the intestinal microbiota and immune function. Probiotics are found naturally in dark chocolate, sauerkraut, yogurt, and most commonly are *Lactobacillus*, *Bifidobacteria*, *Lactococcus*, *Streptococcus*, and *Enterococcus* (Colin et al., 2014). Their primary therapeutic value lies in maintaining commensal microbiota homeostasis, a critical determinant of host physiological functions. They establish ecological interference via the biosynthesis of antimicrobial metabolites, outcompete pathogens, and then inhibit the bacterial toxin biosynthesis and precisely control the host’s immune system (Oelschlaeger Tobias, 2009). They colonize and proliferate in the intestinal tract, fortify epithelial barrier function, adhere to enterocytes, reduce pathogenic microorganisms colonization, and preserve intestinal microbiome homeostasis (Martens et al., 2018). A randomized, double-blind, placebo-controlled trial assessed the influence of *Lactobacillus paracasei-33* (LP-33) enriched fermented milk on the quality of life (QOL) in AR patients. The findings demonstrated that daily intake of LP-33-fermented milk for 1 month significantly and safely enhanced QOL in AR sufferers, suggesting its potential as a complementary treatment option (Fuu et al., 2010). It is noteworthy that most included studies utilized identical probiotic strains with limited microbial diversity coverage. Consequently, a pressing demand exists for additional rigorously designed randomized controlled trials. In addition, probiotic intake may suppress production of allergen-specific IgE and Th2-associated cytokines (IL-4, IL-13; Gyu et al., 2020).

Probiotics can activate adaptive immunity to the extent that the corresponding immune cells produce IgG and IgA antibodies (Murat et al., 2016). In addition, probiotics stimulate Treg cells, CD8 + T cells, and the release of cytokines such as IFN-γ and IL-10 (Frøkiær et al., 2007; Constantin et al., 2025). Antigen-presenting cells exposed to probiotics present innocuous peptides to T cells, which leads to Treg cell activation and subsequent secretion of immunomodulatory cytokines (TGF-β, IL-10) and retinoic acid. This results in the relief of AR. Thus, probiotic intervention represents a promising therapeutic strategy for AR management.

#### 8.2.2 Daily diet

A cohort study indicates that eating more vegetables and yogurt can increase intestinal microbiome diversity (Venter et al., 2021), reduce the risk of AR. Increased intestinal microbiota diversity elevates fecal butyrate concentrations, which serve dual immunomodulatory functions: promoting Treg generation (Roduit et al., 2018). Treg cells produce immunosuppressive cytokines, including IL-10, to alleviate AR. And macrophages can promote the differentiation of Th cells into different subtypes, such as Th1 and Th2, according to the nature of antigens and microenvironmental cytokines. And the specific subtypes corresponding to what kind of antigens and microenvironment need to be further studied. If Th cells differentiate into Th1 cells, then there can be some relief from the symptoms of AR. Dietary advanced glycation end-products (AGEs) decrease intestinal microbial diversity, thereby reducing the production of metabolites that favor fetal immune development (Peter et al., 2017). Although direct studies examining the relationship between AGEs and AR are limited, the mechanism by which AGEs promote allergies through disruption of the intestinal microbiome and immune balance has been elucidated. A study Zhang et al. (2025) demonstrated through multiple experimental models of FA that a significant increase in LPS-producing bacteria belonging to recognized pathogenic and pro-inflammatory groups, such as Alistipes, Desulfovibrio, and Helicobacter, in the high-AGE group. In contrast, bacteria involved in SCFA production, such as *Bifidobacteria*, were depleted in the high-AGE group. Considering that dysbiosis is recognized as a key mechanism in AR pathogenesis and that AR is also Th2-dominant, it is reasonable to hypothesize that dietary AGEs may exacerbate AR through similar pathways—namely, disrupting intestinal microbiota homeostasis and enhancing Th2-type inflammation. This inference offers a novel perspective on understanding the role of modern dietary patterns in the rising prevalence of AR.

Therefore, is it possible to bring relief from AR through purposeful dietary control? Such as eating more fruits and vegetables and reducing the intake of AGEs to reduce the production of pro-inflammatory factors. Therefore, in terms of treatment, regulating the intestinal microbiota by improving the diet and thus alleviating AR is an important intervention. Since diet is directly related to the digestive system and not so much to the respiratory system, and there is a lack of studies related to lung microbiota and diet, the association between pulmonary microbiome and diet still needs further research.

#### 8.2.3 Prebiotics

Prebiotics are selectively fermented food ingredients that modulate intestinal microbial ecology and function. Commonly used are lactofructose, lactose, oligofructose, and oligogalactose (Olveira Articles of Gabriel, 2016), and they can be used as probiotic substitutes or probiotic supports.

A study investigated the effect of dried Maalshahr (a kind of food prepared through barley malt fermentation, and rich in fiber) versus Fexofenadine (a drug known to alleviate AR) in adult AR patients. The clinical status of AR improved in both groups, while nasal congestion, postnasal drip, and headache scores were significantly lower in the Maaloxal group (Derakhshan et al., 2019). This suggests that Maalaxal can be targeted for specific symptoms of AR, such as nasal congestion, postnasal drip, and headache.

Prebiotic compounds are commonly incorporated into infant formula as functional additives. A double-blind, placebo-controlled study assessed the preventive potential of prebiotic oligosaccharides in allergic disorders (Arslanoglu et al., 2012). The cumulative incidence of allergic manifestations was significantly lower in the prebiotic-supplemented group than in the placebo group. The intervention was particularly beneficial in preventing AR.

In conclusion, despite the availability of lactose, sucrose, maalloxan oligosaccharides, etc., in terms of treatment of AR, there is still a paucity of data on other prebiotics for the treatment of AR, and perhaps prebiotic-mediated cytokine regulation represents a novel therapeutic strategy for managing AR.

#### 8.2.4 Antibiotic therapies

Antibiotics are a class of medications that combat bacterial infections through bacteriostatic or bactericidal mechanisms. Unfortunately although they provide critical defense against life-threatening bacterial pathogens, antibiotics may also influence the development of AR antibiotic administration can induce persistent alterations in intestinal microbial communities and host physiology (Anthony et al., 2022). However, long-term administration of antibiotics affects the colonization of beneficial bacterial species and the balance of the ecology of the intestinal microbiota. And this dysbiosis-mediated alteration of the body’s metabolic pattern may affect the immune function of the organism, thereby increasing the incidence of allergic diseases (Shapiro et al., 2022). Therefore, in order to minimize the side effects of antibiotics, An animal study demonstrated that antibiotic exposure may alter the total population and structural composition of the intestinal microbiota in mice (Guo et al., 2019). And that concomitant administration of *Lactobacillus rhamnosus* may help to improve the intestinal microenvironment and correct antibiotic-mediated dysbiosis, thereby minimizing the adverse effects of antibiotics. Therefore, this provides a new way of thinking for antibiotics to ensure the health of the host while treating the disease, thus making up for the shortcomings of antibiotics. Nonetheless, antibiotics remain a viable therapeutic approach for AR at present.

### 8.3 Discussion of treatment limitations

Despite the development of numerous treatment approaches and novel concepts, many challenges remain unresolved. For instance, most current human studies focus on the microbiota as a whole. Since the potential synergistic interactions among bacterial communities remain unclear, and the role of individual bacteria in treating inflammatory diseases like AR is not fully understood, targeted therapies for specific strains lack systematic theoretical underpinnings and clinical experimental research. Given our limited understanding of potential synergistic mechanisms among microbial communities, targeted therapeutic agents for intestinal microbiota-based AR treatment remain scarce. Resolving these challenges would bring significant benefits to human efforts against AR. A brief comparison of the mechanisms of various treatment approaches is presented in Table 2. You can locate the specific position in the text using the reference number.

**Table 2:** Comparison table of mechanisms for various treatment methods.

Treatment	Impact on AR	Mechanism	Reference
FMT	Alleviation	Increase microbial diversity and achieve stable colonization of symbiotic communities	Grehan et al. (2010)
Probiotics	Alleviation	Regulate the composition of the intestinal microbiota and immune function	Colin et al. (2014)
		Activate the adaptive immune system to induce the production of IgG and IgA antibodies by the corresponding immune cells.	Murat et al. (2016)
		Stimulate Treg cells, CD8 + T cells, and the release of cytokines, such as IFN-γ and IL-10	Frøkiær et al. (2007); Constantin et al. (2025)
Dietary Therapy	Alleviation	Intestinal microbiome diversity → promotes butyrate production → enhances Treg cell generation	Venter et al. (2021)
		High AGE levels can significantly increase pro-inflammatory bacterial populations	Zhang et al. (2025)
Prebiotic	Alleviation	Regulate the intestinal microbiome’s ecology and function	Olveira Articles of Gabriel (2016)
Antibiotics	Alleviation	Alter the total abundance and composition of the mouse intestinal microbiota	Guo et al. (2019)
Bacterial lysate	Alleviation	Activation of dendritic cells via Toll-like receptors → promotes restoration of Th1/Th2 balance	Kaczynska et al. (2022)

## 9 Future research directions

Intervention of precise microbiota: With the continuous development of microbiomics and immunology, individualized microbiota intervention therapy will represent an emerging therapeutic paradigm for AR management.

Optimization of animal models: Further refinement of animal models to study the specific mechanisms of action of lung and intestinal microbiota in AR.

Conducting clinical studies: Expanding clinical samples to verify the modulating effects of intestinal microbiota and lung microbiota on AR, with a view to providing new ideas for clinical treatment.

Exploration of Physical Therapy: Physical therapy employs non-pharmacological, non-invasive methods—such as exercise, physical agents (such as sound, light, electricity and heat), or manual techniques—to help patients restore bodily functions, alleviate pain, or improve health conditions. Currently, many researchers are exploring physical therapy approaches to expand treatment options for AR. Although research in this field remains in its early stages, promising results have emerged. For instance, a review summarizing acoustic therapy for AR (Alao et al., 2025) indicates that acoustic treatment shows potential benefits. These include specific sound frequencies potentially reversing biological dysregulation, increasing nitric oxide production, improving mucociliary clearance, and modulating immune responses by activating mechanosensitive pathways and disrupting pathogenic biofilms. Therefore, expanding emerging physical therapies could provide additional avenues for AR treatment.

## 10 Conclusion

In conclusion, the pulmonary and intestinal microbial communities significantly contribute to AR pathogenesis. With the accumulation of published articles in this field, researchers will have more confidence in curing AR as well.

The intestinal-lung axis is a hot topic in the current discussion about the pathogenic link between AR and microbial dysbiosis, and although we are learning more about the association between AR and the microbiota every day, the exact details of this relationship still need to be further investigated, yet key knowledge gaps persist. Are basophils a key bridge to the intestinal-lung axis? Is targeting AR through colony transplantation effective? And the exact mechanism of the microbiota’s effect on AR is a difficult question that we need to focus on at this time.

Although observations from epidemiologic studies have certainly impacted our comprehension of the pathophysiologic link between AR and the microbiota, these findings have not yet led to enhanced clinical interventions. But these enigmas could soon be unraveled. At that time, AR guidelines could incorporate microbiota-based therapies to prevent AR, which impairs normal work. Therefore, more clinical and basic research is still needed to further unravel these complex mechanisms and explore their potential for practical application.

## References

[ref1] Agnieszka K. Martyna K. Paulina C. Kamil J. Andrzej E. (2022). The crosstalk between the gut microbiota composition and the clinical course of allergic Rhinitis: the use of probiotics, prebiotics and bacterial lysates in the treatment of allergic Rhinitis. Nutrients 14:4328. doi: 10.3390/nu14204328, PMID: 36297012 PMC9607052

[ref2] Alao J. O. Lau K. E. M. White D. Lee K. Puli'uvea C. Bartley J. (2025). Acoustic therapy for allergic rhinitis and chronic rhinosinusitis: modulating microbiome, immunity and well-being. Front. Allergy 6:1649031. doi: 10.3389/falgy.2025.1649031, PMID: 40927538 PMC12415040

[ref3] Anthony W. E. Wang B. Sukhum K. V. D'Souza A. W. Hink T. Cass C. . (2022). Acute and persistent effects of commonly used antibiotics on the gut microbiome and resistome in healthy adults. Cell Rep. 39:110649. doi: 10.1016/j.celrep.2022.110649, PMID: 35417701 PMC9066705

[ref4] Arslanoglu S. Moro G. E. Boehm G. Wienz F. Stahl B. Bertino E. (2012). Early neutral prebiotic oligosaccharide supplementation reduces the incidence of some allergic manifestations in the first 5 years of life. J. Biol. Regul. Homeost. 26, 49–59. 23158515

[ref5] Atarashi K. Tanoue T. Oshima K. Suda W. Nagano Y. Nishikawa H. . (2013). Treg induction by a rationally selected mixture of Clostridia strains from the human microbiota. Nature 500, 232–236. doi: 10.1038/nature12331, PMID: 23842501

[ref6] Atarashi K. Tanoue T. Shima T. Imaoka A. Kuwahara T. Momose Y. . (2011). Induction of colonic regulatory T cells by indigenous Clostridium species. Science 331, 337–341. doi: 10.1126/science.1198469, PMID: 21205640 PMC3969237

[ref7] Bayrak D. P. Aksun S. Altin Z. Bilgir F. Arslan I. B. Colak H. . (2018). Its relationship with IL-10, IL-17, TGF-β, IFN-γ, IL 22, and IL-35. Dis. Markers 2018, 1–6. doi: 10.1155/2018/9131432, PMID: 29692871 PMC5859791

[ref8] Bousquet J. Schünemann H. J. Togias A. Bachert C. Erhola M. (2020). Next-generation allergic Rhinitis and its impact on asthma (ARIA) guidelines for allergic rhinitis based on grading of recommendations assessment, development and evaluation (GRADE) and real-world evidence. J. Allergy Clin. Immunol. 145, 70–80.e3. doi: 10.1016/j.jaci.2019.06.049, PMID: 31627910

[ref9] Brough H. A. Liu A. H. Scott S. Kerry M. Abdel D. Brown S. J. . (2015). Atopic dermatitis increases the effect of exposure to peanut antigen in dust on peanut sensitization and likely peanut allergy. J. Allergy Clin. Immunol. 135, 164–170.e4. doi: 10.1016/j.jaci.2014.10.007, PMID: 25457149 PMC4282723

[ref10] Budden K. F. Gellatly S. L. Wood D. L. A. Cooper Matthew A. Mark M. Philip H. . (2017). Emerging pathogenic links between microbiota and the gut–lung axis. Nat. Rev. Microbiol. 15, 55–63. doi: 10.1038/nrmicro.2016.142, PMID: 27694885

[ref11] Charlotte T. Norbert S. Bastian O. (2021). The microbiota in pneumonia: from protection to predisposition. Sci. Transl. Med. 13:eaba0501. doi: 10.1126/scitranslmed.aba0501, PMID: 33441423

[ref12] Clifford L. Antony V. D. (1932). Antony van Leeuwenhoek and his "little animals": being some account of the father of protozoology and bacteriology and his multifarious discoveries in these disciplines. Q. Rev. Biol. 130, 679–680.

[ref13] Colin H. Francisco G. Gregor R. Gibson G. R. Merenstein D. J. Bruno P. . (2014). Expert consensus document: the international scientific Association for Probiotics and Prebiotics consensus statement on the scope and appropriate use of the term probiotic. Nature reviews. Gastroenterol. Hepatol. 11, 506–514. doi: 10.1038/nrgastro.2014.66, PMID: 24912386

[ref14] Constantin T. Maria K. David F. Simon H. Katja D. Oefner P. J. . (2025). A probiotic approach identifies a Treg-centered immunoregulation via modulation of gut microbiota metabolites in people with multiple sclerosis and healthy individuals. EBioMedicine 116, 105743–105743. doi: 10.1016/j.ebiom.2025.105743, PMID: 40359627 PMC12137156

[ref15] Costello E. K. Lauber C. L. Micah H. Noah F. Gordon J. I. Rob K. (2009). Bacterial community variation in human body habitats across space and time. Science 326, 1694–1697. doi: 10.1126/science.1177486, PMID: 19892944 PMC3602444

[ref16] Deepika Alsharari Z. D. Ahmad M. F. Singh J. Yadav M. Kumari A. Kumar A. (2025). Probiotics, and prebiotics: insights on dysbiosis, mechanism, and prevention of lung cancer. Front. Nutr. 12:1624803. doi: 10.3389/fnut.2025.1624803, PMID: 40808835 PMC12345372

[ref17] Derakhshan A. Khodadoost M. Ghanei M. Gachkar L. Hajimahdipour H. Taghipour A. . (2019). Effects of a novel barley-based formulation on allergic Rhinitis: a randomized controlled trial. Endocr Metab Immune Disord Drug Targets 19, 1224–1231. doi: 10.2174/1871530319666190306100611, PMID: 30843497

[ref18] Dickson R. P. Erb-Downward J. R. Martinez F. J. Huffnagle G. B. (2015). The microbiome and the respiratory tract. Annu. Rev. Physiol. 78, 481–504. doi: 10.1146/annurev-physiol-021115-105238, PMID: 26527186 PMC4751994

[ref19] Dong L. Malkowski M. G. (2025). Coupling subunit-specific states to allosteric regulation in homodimeric cyclooxygenase-2. Biochemistry 64, 1380–1392. doi: 10.1021/acs.biochem.4c00821, PMID: 40021482

[ref20] Eggel A. Pennington L. F. Jardetzky T. S. (2024). Therapeutic monoclonal antibodies in allergy: targeting IgE, cytokine, and alarmin pathways. Immunol. Rev. 328, 387–411. doi: 10.1111/imr.13380, PMID: 39158477 PMC11659931

[ref21] Frøkiær L. N. Fink L. H. Zeuthen H. R. Hanne C. B. M. Guido F. (2007). Distinct gut-derived lactic acid bacteria elicit divergent dendritic cell-mediated NK cell responses. Int. Immunol. 19, 1319–1327. doi: 10.1093/intimm/dxm103, PMID: 17951600

[ref22] Fuu W. M. Chuan L. H. Yu W. Y. Hsiang H. C. (2010). Treatment of perennial allergic rhinitis with lactic acid bacteria. Pediatr. Allergy Immunol. 15, 152–158. doi: 10.1111/j.1399-3038.2004.00156.x, PMID: 15059192

[ref23] Gao Y. Long B. Yu S. (2024). Research progress of memory T cells in the pathogenesis of allergic rhinitis. Lin Chuang Er Bi Yan Hou Tou Jing Wai Ke Za Zhi 38, 975–978. doi: 10.13201/j.issn.2096-7993.2024.10.018, PMID: 39390941 PMC11839565

[ref24] Giorgio C. (2010). De amici Mara, Tosca Mariangela and Fuchs Dietmar,.Tryptophan metabolism in allergic rhinitis: the effect of pollen allergen exposure. Hum. Immunol. 71, 911–915. doi: 10.1016/j.humimm.2010.05.017, PMID: 20540982

[ref25] Gregor R. Gadir A. A. Raja D. (2019). Probiotics: reiterating what they are and what they are not. Front. Microbiol. 10:10. doi: 10.3389/fmicb.2019.00424, PMID: 30930863 PMC6425910

[ref26] Grehan M. J. Julius B. T. Leis S. M. Jordana C. Hazel M. Antony W. (2010). Durable alteration of the colonic microbiota by the administration of donor fecal flora. J. Clin. Gastroenterol. 44, 551–561. doi: 10.1097/MCG.0b013e3181e5d06b, PMID: 20716985

[ref27] Guo J.-w. Cheng R.-y. Zhang Y.-j. Zhou W.-x. Wang K. Chen S.-q. . (2019). Alleviating effects of *Lactobacillus rhamnosus* against abnormal intestinalmicrobiota and development induced by ceftriaxone antibiotics in early life. Chin. J. Antibiot. 44, 1107–1113. doi: 10.13461/j.cnki.cja.006630

[ref28] Gyu K. M. Won H. S. Ryun K. H. Jin H. S. Hee C. J. (2020). Probiotic NVP-1703 alleviates allergic Rhinitis by inducing IL-10 expression: a four-week clinical trial. Nutrients 12:1427. doi: 10.3390/nu12051427, PMID: 32429063 PMC7284371

[ref29] Hyun D.-W. Min H. J. Kim M.-S. Whon T. W. Shin N.-R. Kim P. S. . (2018). Dysbiosis of inferior turbinate microbiota is associated with high Total IgE levels in patients with allergic Rhinitis. Infect. Immun. 86:e00934-17. doi: 10.1128/IAI.00934-17, PMID: 29426044 PMC5865026

[ref30] Jacob M. Tal K. (2022). Isakov Naomi Fliss and Maharshak Nitsan,.Clinical efficacy of fecal microbial transplantation treatment in adults with moderate-to-severe atopic dermatitis. Immun. Inflam. Dis. 10:e570. doi: 10.1002/iid3.570, PMID: 34931478 PMC8926506

[ref31] Jin Y. Ping T. Wang Zhongliang X. Xiaoqian Z. X. Meng Xuanyi W. Yong L. X. . (2023). *Staphylococcus aureus* enterotoxin B is a cofactor of food allergy beyond a Superantigen. J. Immunol. 211, 1287–1297. doi: 10.4049/jimmunol.2200549, PMID: 37702994

[ref32] Kaczynska A. Klosinska M. Janeczek K. Zarobkiewicz M. Emeryk A. (2022). Promising immunomodulatory effects of bacterial lysates in allergic diseases. Front. Immunol. 13:907149. doi: 10.3389/fimmu.2022.907149, PMID: 35812388 PMC9257936

[ref33] Kaisar M. M. M. Pelgrom L. R. van der Ham A. J. Yazdanbakhsh M. Everts B. (2017). Butyrate conditions human dendritic cells to prime type 1 regulatory T cells via both histone deacetylase inhibition and G protein-coupled receptor 109A signaling. Front. Immunol. 8:1429. doi: 10.3389/fimmu.2017.01429, PMID: 29163504 PMC5670331

[ref34] Kim W.-G. Kang G.-D. Kim H. I Han M. J. Kim D.-H. (2019). *Bifidobacterium longum* IM55 and *Lactobacillus plantarum* IM76 alleviate allergic rhinitis in mice by restoring Th2/Treg imbalance and gut microbiota disturbance. Benefic. Microbes 10, 55–67. doi: 10.3920/BM2017.0146, PMID: 30465441

[ref35] Koatz A. M. Coe N. A. Alberto C. Alter A. J. (2016). Clinical and immunological benefits of OM-85 bacterial lysate in patients with allergic Rhinitis, asthma, and COPD and recurrent respiratory infections. Lung 194, 687–697. doi: 10.1007/s00408-016-9880-5, PMID: 27117798 PMC7087659

[ref36] Krueger P. D. Goldberg M. F. Hong S. W. Osum K. C. Langlois R. A. Kotov D. I. . (2021). Two sequential activation modules control the differentiation of protective T helper-1 (Th1) cells. Immunity 54, 687–701.e4. doi: 10.1016/j.immuni.2021.03.006, PMID: 33773107 PMC8495663

[ref37] Lebman D. A. Lee F. D. Coffman R. L. (1990). Mechanism for transforming growth factor beta and IL-2 enhancement of IgA expression in lipopolysaccharide-stimulated B cell cultures. J. Immunol. 144, 952–959. doi: 10.4049/jimmunol.144.3.952, PMID: 2295822

[ref38] Lewis G. Wang B. Jahani P. S. Hurrell B. P. Banie H. Aleman G. R. . (2019). Dietary Fiber-induced microbial short chain fatty acids suppress ILC2-dependent airway inflammation. Front. Immunol. 10:10. doi: 10.3389/fimmu.2019.02051, PMID: 31620118 PMC6760365

[ref74] Li Z. Wang S. Pan L. J. Shi L. Zhang Y. L. (2000). Effects of Bifidobacterium full-peptide polysaccharide on IL-6, IL-12, and nitric oxide production in intraperitoneal macrophages from nude mice. Chin. J. Pathophysiol. 16, 58–61.

[ref39] Liu T. Li J. Liu Y. Xiao N. Suo H. Xie K. . (2012). Short-chain fatty acids suppress lipopolysaccharide-induced production of nitric oxide and proinflammatory cytokines through inhibition of NF-κB pathway in RAW264.7 cells. Inflammation 35, 1676–1684. doi: 10.1007/s10753-012-9484-z, PMID: 22669487

[ref40] Mabbott N. A. Donaldson D. S. Ohno H. Williams I. R. Mahajan A. (2013). Microfold (M) cells: important immunosurveillance posts in the intestinal epithelium. Mucosal Immunol. 6, 666–677. doi: 10.1038/mi.2013.30, PMID: 23695511 PMC3686595

[ref41] Maozhen Z. Xi L. Meng Yang L. Haiyan L. K. Pimin G. Tongjie L. . (2024). Probiotics induce intestinal IgA secretion in weanling mice potentially through promoting intestinal APRIL expression and modulating the gut microbiota composition. Food Funct. 15, 4862–4873. doi: 10.1039/d4fo00962b, PMID: 38587236

[ref42] Martens K. Pugin B. Boeck D. I Spacova I. Steelant B. Seys S. F. . (2018). Probiotics for the airways: potential to improve epithelial and immune homeostasis. Allergy 73, 1954–1963. doi: 10.1111/all.13495, PMID: 29869783

[ref43] Maslowski Kendle M. Vieira Angelica T. Ng A. Kranich J. Sierro F. Yu D. . (2009). Regulation of inflammatory responses by gut microbiota and chemoattractant receptor GPR43. Nature 461, 1282–1286. doi: 10.1038/nature08530, PMID: 19865172 PMC3256734

[ref44] Massimiliano M. Maria Z. A. Marta A. Lucia C. A. Catia L. Luca V. . (2020). 16S metagenomics reveals Dysbiosis of nasal Core microbiota in children with chronic nasal inflammation: role of adenoid hypertrophy and allergic Rhinitis. Front. Cell. Infect. Microbiol. 10:458. doi: 10.3389/fcimb.2020.00458, PMID: 32984078 PMC7492700

[ref45] Meng Q. Li P. Li Y. Chen J. Wang L. He L. . (2019). Broncho-vaxom alleviates persistent allergic rhinitis in patients by improving Th1/Th2 cytokine balance of nasal mucosa. Rhinology, 01–459. doi: 10.4193/Rhin19.161, PMID: 31403136

[ref46] Miao P. Jiang Y. Jian Y. Shi J. Liu Y. Piewngam P. . (2023). Exacerbation of allergic rhinitis by the commensal bacterium *Streptococcus salivarius*. Nat. Microbiol. 8, 218–230. doi: 10.1038/s41564-022-01301-x, PMID: 36635572 PMC10062442

[ref47] Murat K. Hakan A. Emin S. Volkan G. Emre K. (2016). The Immunostimulatory effect of lactic acid Bacteria in a rat model. Iran. J. Immunol. 13, 220–228. doi: 10.22034/iji.2016.33410 27671513

[ref48] Nakajima T. Nakanishi S. Mason C. Montgomery J. Leggett P. Matsuda M. . (2014). Population structure and characterization of viridans group streptococci (VGS) isolated from the upper respiratory tract of patients in the community. Ulster Med. J. 82, 164–168. doi: 10.1016/j.poly.2005.04.033 PMC3913407 24505152

[ref49] Natalini J. G. Singh S. Segal L. N. (2023). The dynamic lung microbiome in health and disease. Nat. Rev. Microbiol. 21, 222–235. doi: 10.1038/s41579-022-00821-x, PMID: 36385637 PMC9668228

[ref50] Neff C. Preston R. M. Kathleenl A. Colmb C. Jody D. Nichole N. . (2016). Diverse intestinal bacteria contain putative Zwitterionic capsular polysaccharides with anti-inflammatory properties. Cell Host Microbe 20, 535–547. doi: 10.1016/j.chom.2016.09.002, PMID: 27693306 PMC5113727

[ref51] Oelschlaeger Tobias A. (2009). Mechanisms of probiotic actions - a review. Int. J. Med. Microbiol. 300, 57–62. doi: 10.1016/j.ijmm.2009.08.005, PMID: 19783474

[ref52] Olveira Articles of Gabriel (2016). An update on probiotics, prebiotics and symbiotics in clinical nutrition. Endocrinol. Nutr. 63, 482–494. doi: 10.1016/j.endoen.2016.10.011, PMID: 27633133

[ref53] Ooijevaar Rogier E. Terveer Elisabeth M. Verspaget Hein W. Kuijper Ed J. Keller Josbert J. (2019). Clinical application and potential of fecal microbiota transplantation. Annu. Rev. Med. 70, 335–351. doi: 10.1146/annurev-med-111717-122956, PMID: 30403550

[ref54] Peter K. Smith M. M. Li X.-M. Sampson H. A. (2017). The false alarm hypothesis: food allergy is associated with high dietary advanced glycation end-products and proglycating dietary sugars that mimic alarmins. J. Allergy Clin. Immunol. 139, 429–437. doi: 10.1016/j.jaci.2016.05.040, PMID: 27544741

[ref55] Roduit C. Frei R. Ferstl R. Loeliger S. Westermann P. Rhyner C. . (2018). High levels of butyrate and propionate in early life are associated with protection against atopy. Allergy 74, 799–809. doi: 10.1111/all.13660, PMID: 30390309

[ref56] Roni S. Mika G. Arnon N. Ilan Y. (2019). Fecal Microbiota Transplantation for Treatment of Acute Graft-versus-Host Disease. 1, 28–35. 10.2991/chi.d.190316.002 PMC8432378 34595408

[ref57] Saha J. Goswami R. (2025). Modulation of innate immunity by short-chain fatty acids in probiotic and fecal microbiota transplantation therapies for the treatment of Colon disorders. Probiot. Antimicrob. doi: 10.1007/s12602-025-10807-9, PMID: 41123835

[ref58] Schulthess J. Pandey S. Capitani M. Rue-Albrecht K. C. Arnold I. Franchini F. (2019). The short chain fatty acid butyrate imprints an antimicrobial program in macrophages. Immunity 50, 432–445.e7. doi: 10.1016/j.immuni.2018.12.018, PMID: 30683619 PMC6382411

[ref59] Segal L. N. Blaser M. J. (2014). A brave New World: the lung microbiota in an era of change. Ann. Am. Thorac. Soc. 11, S21–S27. doi: 10.1513/AnnalsATS.201306-189MG, PMID: 24437400 PMC3972973

[ref60] Shapiro D. Kapourchali F. R. Santilli A. Han Y. Cresci G. A. M. (2022). Targeting the gut microbiota and host immunity with a Bacilli-species probiotic during antibiotic exposure in mice. Microorganisms 10:1178. doi: 10.3390/microorganisms10061178, PMID: 35744696 PMC9228267

[ref61] Siddiqui Z. A. Walker A. Pirwani M. M. Tahiri M. Syed I. (2022). Allergic rhinitis: diagnosis and management. Br. J. Hosp. Med. 83, 1–9. doi: 10.12968/hmed.2021.0570, PMID: 35243888

[ref62] Sim S. Choi Y. Yang E. M. Park H. S. (2024). Association between specific IgE to staphylococcal enterotoxin B and the eosinophilic phenotype of asthma. Korean J. Intern. Med. 39, 659–667. doi: 10.3904/kjim.2024.003, PMID: 38986495 PMC11236811

[ref63] Singh V. Kaur R. Kumari P. Pasricha C. Singh R. (2023). ICAM-1 and VCAM-1: gatekeepers in various inflammatory and cardiovascular disorders. Clin. Chim. 548:117487. doi: 10.1016/j.cca.2023.117487, PMID: 37442359

[ref64] Steelant B. Seys S. F. Van Gerven L. Van Woensel M. Farré R. Wawrzyniak P. (2017). Hellings,.Histamine and T helper cytokine-driven epithelial barrier dysfunction in allergic rhinitis. J. Allergy Clin. Immunol. 141, 951–963.e8. doi: 10.1016/j.jaci.2017.08.039, PMID: 29074456

[ref65] Takuho I. Kazuyuki F. Cheng H. Mio U. Tomonori K. Kazuya I. . (2023). Short-chain fatty acids stimulate dendrite elongation in dendritic cells by inhibiting histone deacetylase. FEBS J. 290, 5794–5810. doi: 10.1111/febs.16945, PMID: 37646105

[ref66] Tamasauskiene L. Sitkauskiene B. (2020). Interleukin-22 in allergic airway diseases: a systematic review. J. Interf. Cytokine Res. 40, 125–130. doi: 10.1089/jir.2019.0094, PMID: 31895598

[ref67] Teruyuki S. Wendy H. Hall Jason A. Yi Y. Alessandra C. Gavzy Samuel J. . (2015). An IL-23R/IL-22 circuit regulates epithelial serum amyloid a to promote local effector Th17 responses. Cell 163, 381–393. doi: 10.1016/j.cell.2015.08.061, PMID: 26411290 PMC4621768

[ref68] Tianyi C. Jiarui L. Boxue C. Bin L. Wenzhi Y. Xin Z. . (2024). Polygonatum odoratum polysaccharide ameliorates the allergic airway inflammation through regulating the gut microbiota and inhibiting gut-lung migration of ILC2s. J. Funct. Foods 118:106276. doi: 10.1016/j.jff.2024.106276, PMID: 41262668

[ref69] University of Science and Technology of China, (2010). Zhao Liping regulation of the quorum sensing system of Staphylococcus aureus Al-2.

[ref70] Van Nguyen T. Piao C. H. Fan Y. J. Shin D. U. Kim S. Y. Song H. J. . (2020). Anti-allergic rhinitis activity of α-lipoic acid via balancing Th17/Treg expression and enhancing Nrf2/HO-1 pathway signaling. Sci. Rep. 10:12528. doi: 10.1038/s41598-020-69234-1, PMID: 32719431 PMC7385155

[ref71] Venter C. Palumbo M. P. Glueck D. H. Sauder K. A. O'Mahony L. Fleischer D. M. . (2021). The maternal diet index in pregnancy is associated with offspring allergic diseases: the healthy start study. Allergy 77, 162–172. doi: 10.1111/all.14949, PMID: 34018205 PMC9292464

[ref72] Verma R. Lee C. Jeun E. J. Yi J. Kim K. S. Ghosh A. . (2018). Cell surface polysaccharides of *Bifidobacterium bifidum* induce the generation of Foxp3(+) regulatory T cells. Sci. Immunol. 3:3. doi: 10.1126/sciimmunol.aat6975, PMID: 30341145

[ref73] Von T. Laubach S. (2014). Probiotic administration in early life, atopy, and asthma: a meta-analysis of clinical trials. Pediatrics 134 Suppl 3:S141. doi: 10.1542/peds.2014-1817O, PMID: 25363921

[ref75] Wen X. Xiaoyue D. Longkun D. Yue X. Man Y. Min Z. . (2021). Three main short-chain fatty acids inhibit the activation of THP-1 cells by *Mycoplasma pneumoniae*. Biosci. Biotechnol. Biochem. 85, 923–930. doi: 10.1093/bbb/zbaa110, PMID: 33590852

[ref76] Weronika B. Boutin Rozlyn C. T. Milena S. Brett F. B. (2020). The role of lung and gut microbiota in the pathology of asthma. Immunity 52, 241–255. doi: 10.1016/j.immuni.2020.01.007, PMID: 32075727 PMC7128389

[ref77] Xian Li X. X. Li J. Huang Y. Wang C. Zhang Y. Zhang L. (2022). Direct and indirect costs of allergic and non-allergic rhinitis to adults in Beijing, China. Clin. Transl. Allergy 12:12. doi: 10.1002/clt2.12148, PMID: 35441003 PMC9012971

[ref78] XiangTao L. Li J. Cao J. Li X. Gao Y. Yong X. F. (2020). Dysbiosis of fecal microbiota in allergic Rhinitis patients. Am. J. Rhinol. Allergy 34, 650–660. doi: 10.1177/1945892420920477, PMID: 32340468

[ref79] Xie Z. Eriksen D. B. Johnsen P. R. Nielsen D. S. Frøkiær H. (2025). The effect of microbial metabolites from colonic protein fermentation on bacteria-induced cytokine production in dendritic cells. Biofactors 51:e70007. doi: 10.1002/biof.70007, PMID: 39992073 PMC11849446

[ref80] Xue C. Li G. Zheng Q. Gu X. Shi Q. Su Y. . (2023). Tryptophan metabolism in health and disease. Cell Metab. 35, 1304–1326. doi: 10.1016/j.cmet.2023.06.004, PMID: 37352864

[ref81] Yang W. Cong Y. (2021). Gut microbiota-derived metabolites in the regulation of host immune responses and immune-related inflammatory diseases. Cell. Mol. Immunol. 18, 866–877. doi: 10.1038/s41423-021-00661-4, PMID: 33707689 PMC8115644

[ref82] Yang W. Tianming Y. Huang X. Bilotta A. J. Xu L. Lu Y. . (2020). Microbiota-derived short-chain fatty acids regulation of immune cell IL-22 production and gut immunity. Nat. Commun. Intestinal. 11:4457. doi: 10.1038/s41467-020-18262-6 PMC7478978 32901017

[ref83] Yang G. Wu G. Yao W. Guan L. Geng X. Liu J. . (2022). 5-HT is associated with the dysfunction of regulating T cells in patients with allergic rhinitis. Clin. Immunol. 243:109101. doi: 10.1016/j.clim.2022.109101, PMID: 36029976

[ref84] Yin D. G. He Z. Duan X. Y. Fan F. X. Liao X. B. Wang Q. C. (2019). Effect of probiotic supplementation during pregnancy and infancy in preventing atopic dermatitis in children: a meta analysis. Zhongguo dang Dai Er Ke Za Zhi 21, 82–88. doi: 10.7499/j.issn.1008-8830.2019.01.015, PMID: 30675869 PMC7390177

[ref85] Yiquan Chen Guillemin Gilles J,.Kynurenine pathway metabolites in humans: disease and healthy states. Int. J. Tryptop. Res. 2009, 2009, 2,:2097. doi: 10.4137/ijtr.s2097, PMID: , 22084578 PMC3195227

[ref86] Yuan Y. Wang C. Wang G. Guo X. Shengyu Jiang X. Zuo X. W. . (2022). Airway microbiome and serum metabolomics analysis identify differential candidate biomarkers in allergic Rhinitis. Front. Immunol. 12:771136. doi: 10.3389/fimmu.2021.771136, PMID: 35069544 PMC8766840

[ref87] Zhang Q. Yu G. Jiang Y. Shi H. Yang X. Gao Z. . (2025). Dietary advanced glycation end-products promote food allergy by disrupting intestinal barrier and enhancing Th2 immunity. Nat. Commun. 16:4960. doi: 10.1038/s41467-025-60235-0, PMID: 40436880 PMC12120056

[ref88] Zhao Y. Chen J. Qin Y. Yuan J. Zixian Y. Ma R. . (2025). Linking short-chain fatty acids to systemic homeostasis: mechanisms, therapeutic potential, and future directions. J. Nutr. Metab. 2025:2025. doi: 10.1155/jnme/8870958, PMID: 40761338 PMC12321436

[ref89] Zhou A. Lei Y. Tang L. Hu S. Yang M. Wu L. . (2020). Microbiota: the emerging link to lung homeostasis and disease. J. Bacteriol. Gut 203:203. doi: 10.1128/JB.00454-20, PMID: 33077630 PMC7847545

[ref90] Zhou M.-S. Zhang B. Gao Z.-L. Zheng R.-P. Marcellin D. F. H. M. Saro A. . (2021). Altered diversity and composition of gut microbiota in patients with allergic rhinitis. Microb. Pathog. 161:105272. doi: 10.1016/j.micpath.2021.105272, PMID: 34740809

[ref91] Zhu L. Takashi S. Ruoxi C. Lu M. Qingzhao Z. Lu W. . (2012). Effects of lysed *Enterococcus faecalis* FK-23 on experimental allergic rhinitis in a murine model. J. Biomed. Res. 26, 226–234. doi: 10.7555/JBR.26.20120023, PMID: 23554753 PMC3596073

[ref92] Zhu J. Yamane H. Paul W. (2010). Differentiation of effector CD4 T cell populations. Annu. Rev. Immunol. 28, 445–489. doi: 10.1146/annurev-immunol-030409-101212, PMID: 20192806 PMC3502616

